# Synthesis of New Naphthyl Aceto Hydrazone-Based Metal Complexes: Micellar Interactions, DNA Binding, Antimicrobial, and Cancer Inhibition Studies

**DOI:** 10.3390/molecules26041044

**Published:** 2021-02-17

**Authors:** Fawad Ahmad, Muneera D. F. Alkahtani, Muhammad Babar Taj, Afnan M. Alnajeebi, Seraj Omar Alzahrani, Nouf Abubakr Babteen, Walla Alelwani, Azzah M. Bannunah, Sadia Noor, Rabia Ayub, Syed Ahmad Tirmizi, Heba Alshater

**Affiliations:** 1 Department of Chemistry, Quaid-e-Azam University Islamabad, Islamabad 44000, Pakistan; fawadahmadchemist@yahoo.com; 2 Department of Biology, College of Science, Princess Nourah Bint Abdulrahman University, Riyadh 11675, Saudi Arabia; 3 Department of Chemistry, Islamia University of Bahawalpur, Bahawalpur 63100, Pakistan; 4 Department of Chemistry, University of Sahiwal, Sahiwal 57000, Pakistan; 5 Department of Biochemistry, Faculty of Science, University of Jeddah, Jeddah 80203, Saudi Arabia; Amalnajeebi@uj.edu.sa (A.M.A.); Nababteen@uj.edu.sa (N.A.B.); welwani@uj.edu.sa (W.A.); 6 Department of Chemistry, College of Science, Taibah University, Madinah 42353, Saudi Arabia; szahrani@taibahu.edu.sa; 7 Department of Basic Sciences, Common First Year Deanship, Umm Al-Qura University, Makkah 21955, Saudi Arabia; ambannunah@uqu.edu.sa; 8 Department of Chemistry, Govt. College for Women University Faisalabad, Faisalabad 38000, Pakistan; sadiaa613@gmail.com; 9 Arrhenius Laboratory, Department of Organic Chemistry, Stockholm University, Svante Arrhenius Vag 16C, SE-10691 Stockholm, Sweden; rabiaay1@gmail.com; 10 Department of Forensic Medicine and Clinical Toxicology, Menoufia University, Shbien El-Kom 32511, Egypt; hebaalshater@yahoo.com

**Keywords:** hydrazones, DNA interaction, MALDI, micellar interaction, microbial inhibition, anti-cancer activity

## Abstract

In the present study, naphthyl acetohydrazide (HL) ligand was prepared and used for the synthesis of new six amorphous transition metal (Co(II), Ni(II), Cu(II), Zn(II), Pb(II), Cd(II)) complexes. All the compounds were characterized by elemental analysis, UV-vis, FT-IR, ^1^H- and ^13^C-NMR, and Matrix-Assisted Laser Desorption Ionization (MALDI). The solubilization study was carried out by estimating the interaction between the metal complexes with surfactants viz. sodium stearate (SS) and Cetyltrimethylammonium bromide (CTAB). UV-Visible spectroscopy was employed to determine partitioning and binding parameters, whereas electrical conductivity measurements were employed to estimate critical micellar concentration (CMC), the extent of dissociation, and free energy of micellization. The CT-DNA interaction of synthesized compounds with DNA represents the major groove binding. The synthesized ligand and metal complexes were also tested against bacterial and fungal strains and it has been observed that Cu(II) complex is active against all the strains except Candida albicans, while Cd(II) complex is active against all bacterial and fungal strains except Pseudomonas. Among all compounds, only the Pd(II) complex shows reasonable activity against cervical cancer HeLa cell lines, representing 97% inhibition.

## 1. Introduction

**Figure 1:**
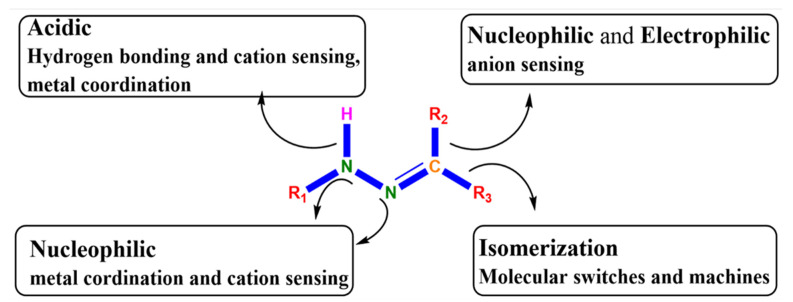
The structural and functional diversity of hydrazine scaffold [10].

Metal-based drugs have a strong therapeutic effect and it has been observed that incorporation of metals not only increases the bioactivity of bioactive ligands but also significantly enhances the pharmacological potential of inactive ligands [1]. Metal coordination is one of the most effectual tactics to plan long-acting or slow-releasing drugs [2,3]. For many years, increasing attention has been paid to the synthesis of hydrazone ligands and their metal-based complexes because of their multipurpose coordination performance [4], chelating scope [5], structural amenability [6], and several other pharmacological properties [6]. Hydrazone with numerous metals has been extensively used as a building block for a wide range of biological treatments [7,8]. Functional diversity of the azomethine group (C-N-N) in hydrazone enables its use in many fields [9]. A close examination of the structure of hydrazone (Figure 1) reveals that it has (i) an imine carbon that has both nucleophilic and electrophilic characters, (ii) amino-type nitrogen’s and nucleophilic imine, (iii) configurational isomerism springing from the native nature of C¼N bond, and (iv) an acidic N–H proton.

The interaction of metal complexes with various surfactants has been previously reported [11,12,13] and such solubilization phenomenon helps to increase the solubility of insoluble or sparingly soluble species in aqueous media. The amphiphilic nature of surfactants helps to accommodate the metal complexes inside the micelles. Such interactions of metal complexes show synergistic interactions of biomembranes and drugs [14,15,16]. Thus, the micelles facilitate solubility of less soluble excipients/additives by controlling their release rates, minimizing degradation, and in turn reducing toxicity. Solubilization or micellar interaction of bioactive compounds is a crucial and decisive role player in many processes at biological and industrial levels. The metal complexes partition themselves between intermolecular bulk aqueous phase and surrounding micellar phase which helps in solubilization [13].

Our previous work [8] on Dehydroacetic acid (DHA) based hydrazone revealed that due to its specific nature of two isomeric keto and enol forms, limited attention is devoted to their metal complexes. There is no literature available on the micellar interaction and biological activity of dehydroacetic acid-based naphthyl aceto hydrazone ligand and its metal complexes. Taken together, due to the status of DHA, hydrazine, and metal incorporated moieties, herein, we designed and synthesized the dehydroacetic acid-based naphthyl aceto hydrazide ligand and its metal complexes to investigate their micellar interaction parameters and their pharmaceutical potential, especially against bacterial and fungal strains.

## 2. Results and Discussion

**Figure 2:**
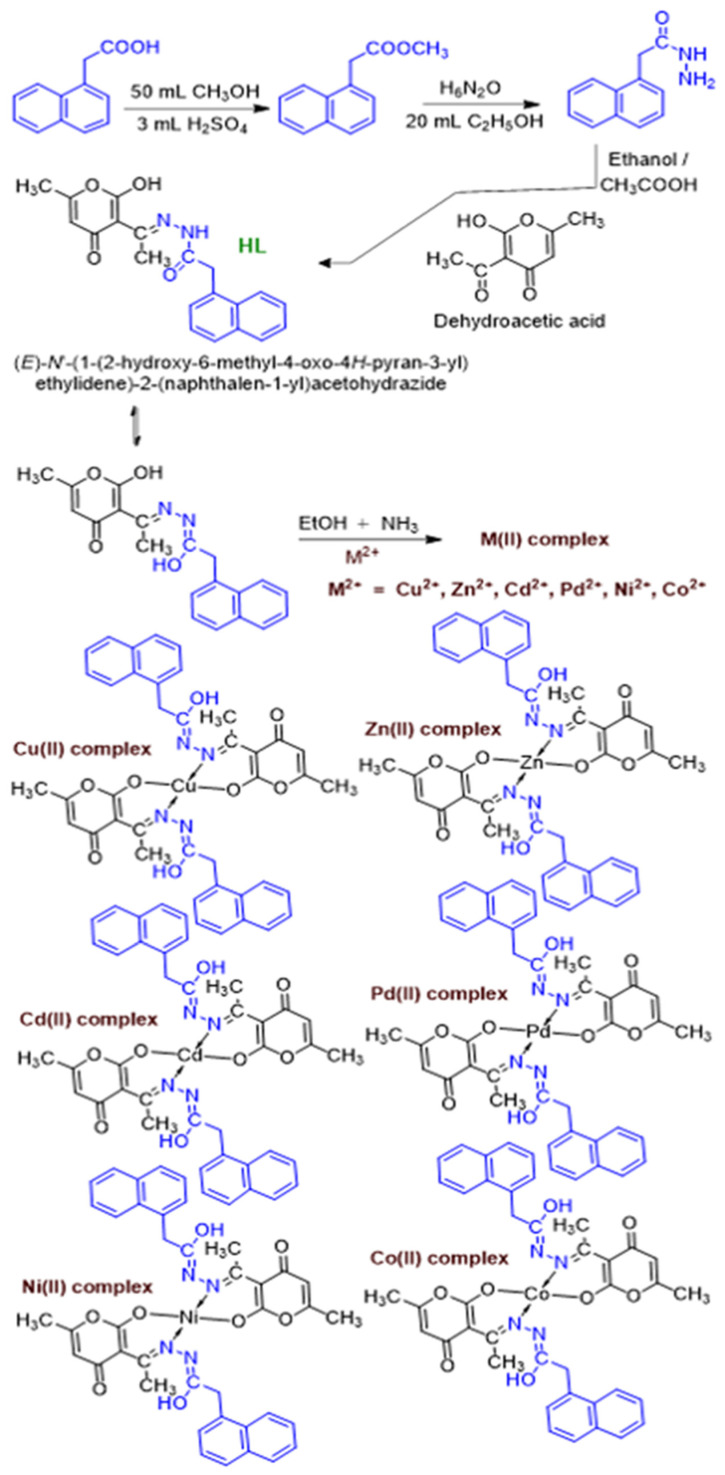
Synthesis of naphthyl hydrazone ligand and their metal (M) derivatives.

The characterization of ligand and metal complexes (Figure 2) is explained as follows. 

### 2.1. FT-IR Spectroscopy

The FT-IR spectra of synthesized ligands represent its formation by indicating the peak at 3275 cm^−1^ due to N-H stretching frequency followed by a weak signal of hydrogen-bonded O-H at 2977 cm^−1^. The C=O stretching frequency appeared at 1702 cm^−1^ and a sharp signal at 1654 cm^−1^ depicts hydrazone based immine C=N. Finally, two peaks at 1511 and 1466 cm^−1^ reflect aromatic stretching frequencies [17]. The comparative structural elucidation of novel metal (Co, Ni, Cu, Zn, Pd, Cd) complexes of synthesized ligand depict the differences in the FT-IR spectra. The FT-IR spectrum of Co(II) complex showed a weak stretching band of hydrogen-bonded O-H at 3142 cm^−1^ and C-H stretching appeared at 3015 cm^−1^. The variation in the cases of C=O and C=N stretching bands at 1693 and 1619 cm^−1^ indicates the formation of a complex. Besides these, the new peaks at 489 cm^−1^ and 568 cm^−1^ indicate the Co-N and Co-O bands, respectively [18]. In the FT-IR spectrum of paramagnetic Ni(II) complex N-H stretching band appears with a broad peak at 3437 cm^−1^ followed by C=O and C=N stretch at 1676 and 1619 cm^−1^ respectively. The two bands at 440 cm^−1^ and 570 cm^−1^ characterize Ni-N and Ni-O respectively [18]. The Cu(II) complex, due to its paramagnetic nature, was characterized only by FT-IR spectroscopy where O-H stretching frequency shifted from 3275 to 3208 cm^−1^ followed by more shifting of stretching frequency bands of C=O and C=N at 1702, 1684 cm^−1^ to 1680 and 1654 cm^−1^ respectively as compared to the ligand. The two bands at 538 cm^−1^ and 480 cm^−1^ represent Cu-N and Cu-O respectively [19]. FT-IR spectrum of the Zn(II) complex showed an absence of O-H stretching frequency with appearing of N-H band at 3147 cm^−1^ followed by the clear difference of C=O and C=N stretching frequencies at 1692, 1625 cm^−1^, respectively The new Zn-N and Zn-O bands appear at 488 cm^−1^ and 527 cm^−1^, respectively [19]. FT-IR spectrum of the Pd(II) diamagnetic complex showed an absence of O-H stretching frequency with the appearance of N-H band at 3310 cm^−1^ followed by C=O and C=N stretching frequencies at 1689, 1563 cm^−1^, respectively. The new Pd-O and Pd-N bands appear at 492 cm^−1^ and 437 cm^−1^, respectively [20]. The FT-IR spectrum of Cd(II) complex displayed the absence of O-H stretching band and showed the weak band of hydrogen-bonded N-H at 3142 cm^−1^, followed the appearance of C-H stretch at 2962 cm^−1^. This may be due to the tautomerism in complex structure as depicted in Figure 1. The C=O and immine C=N stretch appeared at 1676 and 1624 cm^−1^ respectively. The new bands at 482 cm^−1^ and 434 cm^−1^ successively represent Cd-N and Cd-O, respectively [21,22] as shown in Table 1.

### 2.2. Multi-Nuclear (^1^H, ^13^C) NMR and MALDI Analysis

The results of ^1^H-NMR spectrum of a ligand, when recorded in DMSO-d6, confirmed its structure. For example, the presence of various respective shifts, singlet of hydroxyl proton at 16.14 ppm and singlet of NH proton bonded to carbonyl group give a chemical shift at 11.54 ppm. The seven naphthyl aromatic protons appeared as multiplets in the region between 8.10–7.47 ppm. An olefinic singlet due to one proton was observed at a chemical shift of 5.85 ppm. Moreover, two methylene protons with a singlet of azomethine and doublet olefinic methyl protons appear at 4.16, 2.10, 2.56, 2.50 ppm (*J* = 2.51) respectively. Further investigation of the structure via ^13^C-NMR illustrates that azomethine and olefin linked carbon nuclei of CH_3_ appeared at 17.24 and 19.71 ppm. Active methylene signals at 37.77 ppm and the two carbonyl carbon nuclei in the ligand showed a couple of signals at 168.33 and 168.53 ppm, respectively. The naphthyl carbon nuclei appeared in the region between 124.52–133.82 ppm and pyran ring carbon nuclei are characterized at 95.10 and 105.96 ppm. Finally, azomethine carbon nuclei appeared at 181.60 ppm. The structure of ligand was further supported and confirmed by MALDI spectrum where *m/z* = 351 represents the molecular ion peak which is also acting like base peak based on its stability. 

The formation of the Zn(II) complex was further supported by ^1^H NMR in which the major difference is the disappearance of OH proton and shifting of NH singlet to downfield at 5.51 ppm. Besides these, there is a clear shift of an olefinic proton from 5.85 to 4.17 ppm in comparison with the ligand. Similarly, the ^13^C-NMR spectrum of the complex is entirely different from the ligand, for example, carbon nuclei of CH_3_ appear at 19.39 and 20.18 ppm, azomethine carbon nuclei displayed a signal at 170.47 ppm while the carbonyl carbon appeared at 179.21 ppm. The structure was further confirmed by MALDI spectrum where *m*/*z* = 762.00 is a molecular ion peak while after fragmentation *m*/*z* = 413.54 represents the base peak.

Similarly, the ^1^H-NMR of Pd(II) complex supported its formation by the disappearance of OH proton and appearance of NH singlet at 5.92–5.85 ppm. Moreover, olefinic protons gave two signals at 4.15 to 4.08 ppm. The CH_2_ (methylene) protons appeared at 3.83 ppm. Similarly, the ^13^C-NMR spectrum of the complex is entirely different from the ligand regarding carbon nuclei of CH_3_ split up into four signals appearing between 19.72–17.23 ppm. Azomethine carbon nuclei gave a signal at 174.33 ppm while the carbonyl carbon appeared at 181.61 ppm. Finally, olefinic carbon nuclei presented its peak at 95.10 ppm. Moreover, the formation of the complex was further confirmed by MALDI spectrum where *m*/*z* = 805.34 is a molecular ion peak while after fragmentation *m*/*z* = 413.54 represents the base peak which is maybe due to the removal of Pd(L). 

The ^1^H NMR of Cd(II) complex showed the naphthyl protons between 8.07–7.39 and NH protons connected with C=O gave a shift of 5.54 ppm. The olefinic proton appeared at 4.12 ppm with the addition of CH_2_ protons and CH_3_ protons between 3.35 ppm and 2.01 ppm in the spectrum. The structural elucidation was further supported by ^13^C-NMR in which carbon of CH_3_ appeared at 20.63 and 19.44 ppm. Pyran ring carbon nuclei appear at 108.18 ppm closer to naphthyl carbon region between 124–133.76 ppm. Azomethine carbon nuclei showed a peak at 159.91 ppm. The carbonyl carbon appeared at 170.34 ppm. Finally, the structure was confirmed by MALDI, where *m*/*z* = 813 is behaving dually as a molecular ion peak as well as base peak due to its good stability. 

The Co(II), Ni(II), and Cu(II) complexes were only characterized by FT-IR and MALDI spectrometry due to their paramagnetic nature. The structure of Co(II), Ni(II), and Cu(II) complexes were confirmed by MALDI spectrometer with an instrumental error *m/z* = ±2. The Co(II) complex was also confirmed by MALDI spectra where *m*/*z* = 758.26 indicates the molecular ion peak and after the fragmentation of one ligand, the complex indicates its base peak at *m*/*z* = 409.9 with good stability. In the case of Cu(II) complex, the molecular ion peak appeared at *m*/*z* = 762.45 with less stability and a base peak at *m*/*z* = 413.18 which mainly represents the detachment of one ligand from the complex after a possible fragmentation. The MALDI spectrum of Ni(II) complex showed a molecular ion peak at *m*/*z* = 758.26 with good stability while a base peak appeared at *m*/*z* = 407.31.

### 2.3. UV-Vis Studies of Ligand and Metal Complexes 

UV-vis absorption spectra of ligand and metal complexes separately were studied by taking a varied concentration of synthesized compounds. The recorded UV-vis spectra represent a good illustration for comparative data between non-conjugated ligands and conjugated ligands. The synthesized ligand of concentration, (0.009 g/10 mM), (0.23 mL/2.3 mL DMSO) showed an intense absorption band π–π* with λ_max_ value of 325 nm The observed higher shift in value was due to two main factors, (1) The resonance and slight conjugation present only on one terminal of synthesized ligands, (2) It may be due to pyran ring involved in resonance and keto-enol tautomerism, and one more band at lower frequency was due to n–π* arising by non-bonding electrons of carbonyl and azomethine linkage as a charge transfer band of a wavelength having λ_max_ value at 294 nm (A = 1.026). Besides these, there were some other bands with a lower value of λ_max_. There is a clear difference in the shift between the ligand and synthesized metal complexes, which mainly corresponds to L→M charge transfer bands. The spectral data of the Co(II) complex, (0.019 g/10 mM), (0.22 mL/2.3 mL DMSO) vary in comparison to a free ligand and displayed λ_max_ values at 322 nm (A = 1.137) and 282 nm (A = 1.416). The d-d transitions in these cases were too weak to be observed. Similarly, spectra of Ni(II) having concentration, (0.018 g/10 mM), (0.12 mL/2.3 mL DMSO) was presented by one intense and one weak band and showed λ_max_ values of 337 nm (A = 1.123) and 283 nm (A = 1.138), respectively. The electronic spectra of Cu(II) complex having concentration, (0.019 g/10 mM), (0.205 mL/2.3 mL DMSO) represented a broad absorption band at 337 nm (A = 1.067), which is due to LMCT and only difference of 12 nm in the shift was observed in comparison to free ligand. The electronic spectra of Pd(II) complex having concentration, (0.019 g/10 mM), (0.14 mL/2.3 mL DMSO) showed an absorption band having λ_max_ at 330 nm (A = 1.106), which could be assigned as charge transfer transition. As Zn(II) complex having concentration, (0.019 g/10 mM), (0.2 mL/2.3 mL DMSO) and Cd(II) complex having concentration, (0.020 g/10 mM), (0.2 mL/2.3 mL DMSO) complexes were diamagnetic in nature, so in these cases d-d transitions were forbidden. For such types of complexes, the electronic transition occurred only because of ligand, but there are observable shifts in λ_max_ values at 341 nm (A = 1.226) and 359 nm (A = 1.131) respectively from free ligand, which indicates co-ordination (Figure S1 and Table S1).

### 2.4. Micellar Interaction of Metal Complexes

#### 2.4.1. UV-Vis Spectroscopic Measurements

The partition and binding attributes and their corresponding trends were estimated from the spectroscopic analysis of complex molecules with the surfactants. The maximum absorbance of the synthesized complexes was recorded in DMSO and water, initially due to less solubility in pure water (Table 1) and thereafter these values were selected for the subsequent micellar study. The bathochromic shift was observed to various extents in all cases which verified the interaction of complexes with surfactants due to the predominant host-guest type association. Higher magnitude of maximum absorbance in the case of CTAB indicated deep penetration within micelles compared to SS. The involvement of structural attributes of all complexes changed with a molar concentration of surfactants from pre-micellar to the micellar region (critical micellar concentration CMC); however, in the post-micellar region, the microenvironment became steadier and no further change in absorbance was observed. 

**Figure 3:**
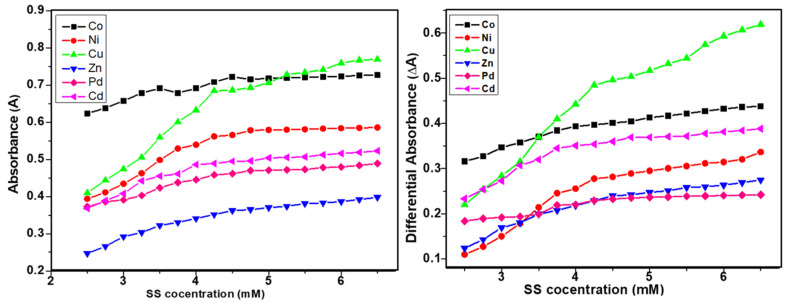
Plots of simple and differential absorbance vs. sodium stearate (SS) concentration.

**Figure 4:**
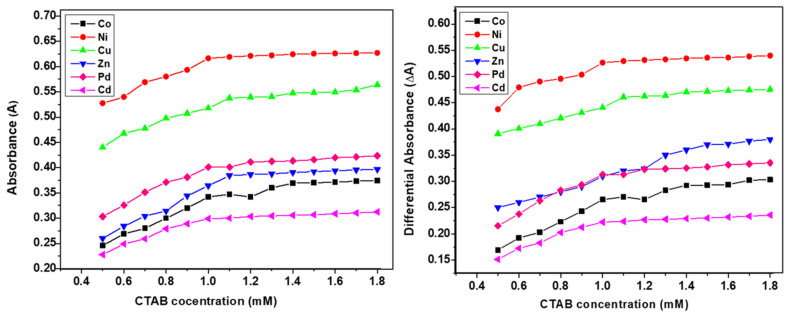
Plots of simple and differential absorbance vs. Cetyltrimethylammonium bromide (CTAB) concentration.

The differential spectroscopy provides a comprehensive interpretation of the presence and extent of interactions of surfactants and additives, drugs, or metal complex molecules. The similar trends in simple and differential absorption were seen and the gradual increase in absorbance until CMC was observed due to the inclusion of metal complex molecules in micellar media of SS and CTAB (Figure 3 and Figure 4). The partition and binding trends were calculated using expressions given in Equations (1)–(5) (see experimental section) and results are tabulated in Table 2.

#### 2.4.2. Electrical Conductivity Measurements

**Figure 5:**
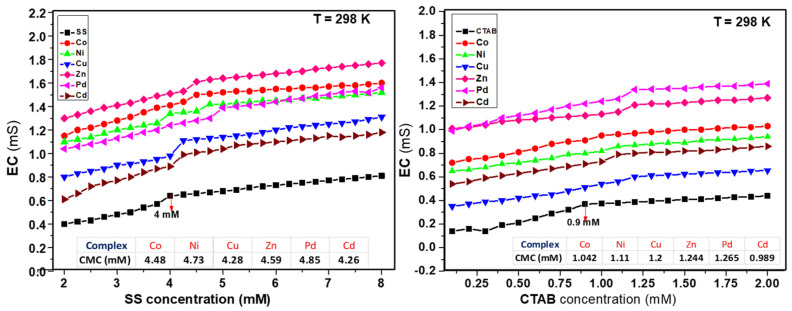
Plots of electrical conductivity vs. concentration of surfactants.

The change in electrical conductivity is a useful indication of interaction between surfactant and complex molecules. The plots in Figure 5 are representing the electrical conductivity against the concentrations of SS and CTAB at 298 K. The involvement of both surfactants enhanced the numerical value of the CMC compared to the pure surfactants at ambient temperature. Significant variation in conductivity was observed after attaining the CMC of the aqueous solutions of complexes in the presence of surfactant. The point of intersection in the conductivity/concentration plots in pre-micellar and post-micellar regions gave us the value of CMC as shown in Figure 5 for SS and CTAB.

Micellization is a complicated process and there is a critical relationship between surfactant’s concentration and formation of micelles. The real mechanistic details of this process are still under debate. The negative values of the free energy and change of micellization (Δ*G*^°^*_m_*) of both micellar media revealed that this was a spontaneous and stable process. Higher negative values of Δ*G*^°^*_m_* showed the higher solubilization of complexes in the micelles of CTAB in comparison to SS. From the increase in the CMC seen in the experimental outcomes, values were observed, and complexes interacted with both ionic surfactants differently. Moreover, the electrostatic forces and the nature of metal decided their positions in corresponding micellar systems. The micellar and thermodynamic attributes are presented in Table 3.

#### 2.4.3. Comparative Interaction of Metal Complexes in Both Surfactants 

Micellization or solubilization is a dynamic process and it allows excipient/additive molecules to reside for different periods in the micellar interior and its outer surface; thus, we witnessed random or fluctuating absorbance values. The degree of solubilization of the complexes in micellar media and their plausible loci in micellar regions are directly influenced by the affected temperature, chemical, structural nature of complex molecules, the concentration of surfactants and metal complexes. Thus, the movement and mechanism of solubilization were governed by partitioning of metal complexes between micellar and aqueous phases.

The higher value of *K_x_* of Ni(II) in CTAB revealed its higher tendency to transfer towards micellar media than SS and is positioned near the peripheral region of micelles. The value of partition coefficient *K_x_* (22.8 × 10^3^ dm^3^/mol) in the micelles of CTAB was higher as compared to SS micelles (1.76 × 10^3^ dm^3^/mol). The negative value of Δ*G_p_* (−34.81 and −28.46) confirmed the stability and spontaneity of the system. The negative values of energies of binding and portioning indicated that the interaction of metal complexes in the micellar media was spontaneous and higher the value of *K_x,_* the more was solubilization. The values of all complexes in both micellar media (as summarized in Table 3) indicates the higher solubilization of complexes in the micelles of CTAB as compared to SS.

**Figure 6:**
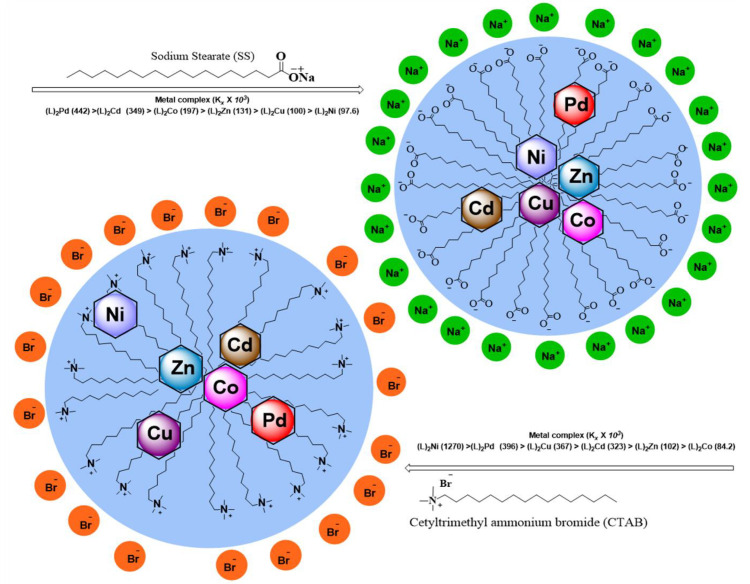
Partitioning behaviour of M(II) complexes in the micelles of SS and CTAB.

Figure 6 shows how all metal complexes were positioned in different zones of micellar media depending on their portioning tendencies. The partitioning study shows that complexes were distributed between aqueous and micellar medium depending on their interaction with the micelles. The structure breaking effect of metal complexes increased the values of CMC in all cases in comparison to pure surfactants (SS 4 mM and CTAB 0.9 mM). The values of CMC of SS shifted to 4.56 mM for Co(II) complex; 4.69 mM for Ni(II) complex; 4.34 mM for Cu(II) complex; 4.62 mM for Zn(II); 4.8 mM for Pd(II) complex, and 4.19 mM for Cd(II) complex. Whereas in case of CTAB, the values of CMC shifted to 1.03 mM for Co(II) complex; 1.09 mM for Ni(II) complex; 1.17 mM for Cu(II) complex; 1.19 mM for Zn(II) complex; 1.23 mM for Pd(II) complex, and 0.995 mM for Cd(II) complex as determined by UV-vis absorbance vs. concentration plots. The values of CMC were obtained from the intersectional point absorbance-concentration plot in the pre-micellar and post-micellar regions. The continuously rising simple differential absorption with the increasing concentrations of SS and CTAB was attributed to incessant and uninterrupted incorporation of complex molecules in micelles. The values of CMC obtained from both techniques were in close agreement. The slight difference in CMC values represents a range of concentration and separate working principles of both techniques. 

### 2.5. DNA Interaction Study

**Figure 7:**
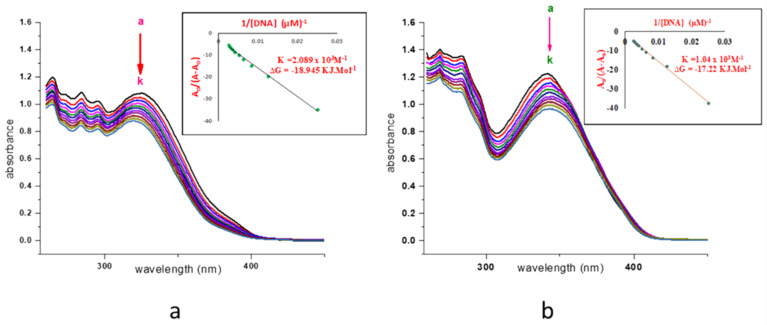
(**a**) DNA Binding study of ligand (**b**) DNA Binding study of Zn(II) complex. (a–k, represent addition of DNA from 0.1 mL to 1.1 mL).

The ligand displayed the two prominent λ_max_ at 325 nm which indicates π→π* transition. With the successive addition of 0.1 mL (0.9 µM) DNA in a complex solution of (0.23 mL in 2.3 mL DMSO), only the intercalative modes were obtained due to hydrogen bonding. The λ_max_ at 325 nm leads to blue shift (hypsochromic) of 5.5 nm from 325 nm→319.5 nm with a decrease in absorbance from 1.083→0.877 and it confirmed intercalating or groove mode with DNA. The binding constant, as well as Gibb’s free energy (Δ*G* = RTlnK), was also calculated. The cobalt and nickel complexes exhibited λ_max_ towards lower wavelength, at 318.5 nm and 341 nm from 322 nm and 345 nm respectively, with an average difference of 3.25 nm and this indicates a negligible binding with DNA. Cu(II) complex without DNA gives its λ_max_ at 337 nm comparative to ligand hydrazone on successive addition of 0.1–0.9 mL DNA and represented a shift of λ_max_ up to 328.5 nm with a difference of only 8.5 nm which confirms the interaction via intercalative mode. The Zn(II) complex displayed entirely different behavior in interaction studies with DNA. Free complex prepared by dissolving 0.2 mL in 2.2 mL DMSO to get absorbance close to 1, as with all other complexes, represents λ_max_ at 341.5 nm. The redshift was observed toward higher λ_max_ with the decrease in absorbance after the first three additions from 0.1→0.3 mL. Furthermore, λ_max_ shifted back to 342 nm after two more additions. The continual increase of the next five additions leads to λ_max_ of 345 nm, which is a shift of only 4.5 nm. This behavior of the Zn(II) complex may be due to weak intercalative mode of interaction with DNA. This could be due to the coupling of the π* orbital of the intercalators with the π orbital of the base pairs which decrease the π-π* transition probabilities and consequently lead to hypochromism [23]. Pd(II) complex with a minute difference displayed the same results as Zn(II) complex with a blue shift of only 4.5 nm. Cd(II) complex interacted DNA by showing an irregular trend with a redshift from 359→363 nm (Figures S2–S7). The difference in activity of metal complexes is due to the different nature of metals present in it. It may also be attributed to the purity of the compound. The interaction mood of DNA with ligand and zinc complex is illustrated in Figure 7.

### 2.6. Anti-Bacterial and Anti-Fungal Studies

It has been observed that Hydrazone based ligand, Co(II) and Pd(II) complexes only show zone of inhibition (mm/100 µL) against Pseudomonas and Candida albicans respectively. Ni(II) and Zn(II) complexes are found to be inactive against all bacterial and fungal strains. Cu(II) complex is active against all the strains except candida albicans while Cd(II) complex is active against all bacterial and fungal strains except pseudomonas (Table 4). The activity of Cu(II) complex could be due to the redox nature of Cu^2+^ ions [24].

### 2.7. MTT Based Cell Viability

The cell viability test was performed in the form of triplicate. From the series of complexes examined herein only Cu(II) and Pd(II) complexes were found to be active against cervical HeLa cell lines in comparison with standard Doxorubicin (positive control). The comparative results (Table 5) represent the activity of the ligand and the M(II) complexes. The activity of Cu(II) and Pd(II) hydrazone complexes could be due to the presence of Cu(II) and Pd(II) metals. Further research is needed to investigate the mechanism of inhibition of Cu(II) and Pd(II) complexes.

## 3. Materials and Methods

All the chemicals used for synthesis were of analytical grade. Metal salts were purchased from Merck. Dehydroacetic acid (DHA) was obtained from Aldrich. Ethanol, Methanol, THF, and DMSO were used as it without further purification and were purchased from Sigma Aldrich. The progress of the reaction was monitored through TLC by using a combination of hexane-ethyl acetate (7:3) solvents. Melting points were determined on Gallonkamp melting point apparatus using open capillaries and were uncorrected. The solution conductivity measurements were performed by dissolving the complexes in DMSO with 10^−3^ M concentration and their molar conductivities were measured at room temperature. Infrared spectra were recorded on Jasco FT-IR-420 spectrophotometer. The multinuclear (^1^H and ^13^C) NMR were recorded on Bruker AM-250 spectrometers in DMSO solution using TMS as an internal standard. UV-vis absorption spectra and DNA binding studies were recorded on UV-vis Spectrophotometer Shimadzu UV-1700. Mass spectra were recorded by using low mass MALDI standard mixture under 6000 *m*/*z* Ultra Flex Analysis placing 1 µL spot of DHB (2,4-dihydroxybenzoic acid) as matrix and 1 µL of the standard mixture in acetonitrile.

### 3.1. General Procedure for the Synthesis of (E)-N′-(1-(2-Hydroxy-6-Methyl-4-oxo-4H-Pyran-3-yl Ethylidene)-2-(Naphthalen-1-yl)Acetohydrazide Ligand (HL)

(i) Synthesis of methyl 2-(naphthalen-1-yl)acetate

First, the mixture of 2-(naphthalen-1-yl) acetic acid (5 g, 0.03 moles), 50 mL methanol and a catalyst (3 mL conc. H_2_SO_4_) was refluxed for twelve hours to yield a methyl 2-(naphthalen-1-yl)acetate. The basic workup was followed by extraction with ethyl acetate and further purification through column chromatography which results in the successful separation of powdered product.

(ii) Synthesis of 2-(naphthalen-1-yl)acetohydrazide

The ethanolic (20 mL) mixture of methyl 2-(naphthalen-1-yl)acetate and hydrazine hydrate (1:1) was refluxed for six hours and then poured into ice. The formed fluffy solid was washed with water and ethanol to give 90% of 2-(naphthalen-1-yl)acetohydrazide.

(iii) Synthesis of (*E*)-*N*′-(1-(2-hydroxy-6-methyl-4-oxo-4H-pyran-3-yl ethylidene)-2-(naphthalen-1-yl)acetohydrazide ligand (HL)

Finally, the ethanolic solution of 2-(naphthalen-1-yl)acetohydrazide and an ethanolic solution of Dehydroacetic acid, DHA (1:1 ratio) were refluxed for four hours in the presence of five drops of glacial acetic acid as a catalyst. (E)-N’-(1-(4-hydroxy-6-methyl-2-oxo-2H-pyran-3-yl)ethylidene)-2-(naphthalen-2-yl)acetohydrazide (HL) appeared in the form of fluffy shiny white ppt. (Figure 1) Yield: 83%; m.p. 199–203 °C. Anal. Calcd. for C_20_H_18_N_2_O_4_ (*Mw* = 350.4 gmol^−1^). C, 68.56; H, 5.18; N, 8.00%. Found: C, 67.90; H, 5.55; N, 7.81%. FTIR (KBr, cm^−1^): 3275 (O-H str.), 2997 (O-H str.), 1702 (C=O str.), 1654 (C=N str.), 1511, 1466 (aromatic str.) ^1^H-NMR (DMSO-*d*_6_, 300 MHz, *δ*/ppm): 16.14 (1H, s, OH), 11.54 (1H, s, NH), 8.10–7.47 (7H, s, naphthyl), 5.85 (1H, s, olefinic), 4.16 (2H, s, CH_2_), 2.56 (3H, s, CH_3_ olefinic), 2.10 (3H, s, CH_3_ azomethine); ^13^C-NMR (DMSO-*d*_6_, 75 MHz, *δ*/ppm): 181.60, 168.33, 163.62, 162.75, 133.82–124.52, 105.96, 95.10, 37.77, 19.71, 17.24. λ_max_ (nm): 325. *m*/*z*: 351

### 3.2. Synthesis of Metal Complexes

To an ethanolic solution (5 mL) containing 2.0 mmol of HL, an ethanolic solution (5 mL) of metal acetates or nitrates (1.0 mmol) were added dropwise with constant stirring. The triethylamine was added to facilitate the binding of metal with co-ordination site of ligand and stirred at room temperature for about 3h. The precipitated metal complex was filtered off and washed with hot ethanol, petroleum ether, and finally air-dried to form amorphous solid (Figure 3). Unfortunately, no single crystals were obtained, and characterization is reduced to elemental analysis, UV-vis, FT-IR, multinuclear (^1^H and ^13^C) NMR, and MALDI mass spectrometry.

(iv) ((*E*)-*N*’-(1-(4-hydroxy-6-methyl-2-oxo-2H-pyran-3-yl)ethylidene)-2-(naphthalen-2-yl)acetohydrazide)2Co.

Pale orange solid. Yield: 61%; m.p. >350 °C. Anal. Calcd. for C_40_H_34_N_4_CoO_8_ (*Mw* = 757.67 gmol^−1^). C, 63.41; H, 4.52; N, 7.39%. Found: C, 62.47; H, 4.38; N, 6.85%. FT-IR (KBr, cm^−1^): 3142 (O-H str.), 3015 (C-H str.), 1693 (C=O olefinic str.), 1619 (C=N), 1428, 1410 (aromatic str.), 568 (Co-N), 457 (Co-O). λ_max_ (nm): 322. *m*/*z*: 758.26

(v) ((*E*)-*N*’-(1-(4-hydroxy-6-methyl-2-oxo-2H-pyran-3-yl)ethylidene)-2-(naphthalen-2-yl)acetohydrazide)2Ni.

Orange solid. Yield: 61%; m.p. 321 °C. Anal. Calcd. for C_40_H_34_N_4_NiO_8_ (*Mw* = 757.5 gmol^−1^). C, 63.43; H, 4.52; N, 7.40 %. Found: C, 63.40; H, 4.55; N, 7.30 %. FT-IR (KBr, cm^−1^): 3437 (N-H str.), 3035 (C-H str.), 1676 (C=O olefinic str.), 1619 (C=N), 1455, 1428 (aromatic str.), 456 (Co-N), 429 (Ni-O). λ_max_ (nm): 345. *m*/*z*: 758

(vi) ((*E*)-*N*’-(1-(4-hydroxy-6-methyl-2-oxo-2H-pyran-3-yl)ethylidene)-2-(naphthalen-2-yl)acetohydrazide)2Cu.

Light green solid. Yield: 65%; m.p. 330 °C. Anal. Calcd. for C_40_H_34_N_4_CuO_8_ (*Mw* = 762.28 gmol^−1^). C, 62.86; H, 4.75; N, 7.33%. Found: C, 62.94; H, 4.69; N, 7.35%. FT-IR (KBr, cm^−1^): 3208 (O-H str.), 3048 (C-H str.), 1680 (C=O str.), 1654 (C=N), 1459, 1428 (aromatic str.), 480 (Cu-N), 427 (Cu-O). λ_max_ (nm): 337. *m*/*z*: 762.4

(vii) ((*E*)-*N*’-(1-(4-hydroxy-6-methyl-2-oxo-2H-pyran-3-yl)ethylidene)-2-(naphthalen-2-yl)acetohydrazide)2Zn.

White solid. Yield: 84%; m.p. >350 °C. Anal. Calcd. for C_40_H_34_N_4_ZnO_8_ (*Mw* = 762.1 gmol^−1^). C, 62.88; H, 4.79; N, 7.33%. Found: C, 62.90; H, 4.70; N, 7.45%. FT-IR (KBr, cm^−1^): 3147 (N-H str.), 2961 (C-H str.), 1692 (C=O str.), 1625 (C=N), 1456, 1442 (aromatic str.), 488 (Co-N), 454 (Co-O). ^1^H-NMR (DMSO-*d*_6_, 300 MHz, *δ*/ppm): 7.99–7.25 (14H, m, 2× naphthyl), 5.51 (2H, s, 2× NH), 4.17 (2H, s, olefinic), 4.12–3.86 (4H, m, 2× CH_2_), 3.37–250. (6H, m, 2× CH_3_ olefinic), 2.43–2.01 (6H, s, 2× CH_3_ azomethine); ^13^C-NMR (DMSO-*d*_6_, 75 MHz, *δ*/ppm): 179.21, 170.47, 164.60, 162.72, 133.75-124.29, 107.60, 96.85, 20.18, 19.39. λ_max_ (nm): 337. *m*/*z*: 762

(viii) ((*E*)-*N*’-(1-(4-hydroxy-6-methyl-2-oxo-2H-pyran-3-yl)ethylidene)-2-(naphthalen-2-yl)acetohydrazide)2Pd.

Pale yellow solid. Yield: 74%; m.p. 320 °C. Anal. Calcd. for C_40_H_34_N_4_PdO_8_ (*Mw* = 805.5 gmol^−1^). C, 59.67; H, 4.26; N, 6.96%. Found: C, 60.57; H, 4.30; N, 7.25%. FT-IR (KBr, cm^−1^): 3310 (N-H str.), 3182 (C-H str.), 1689(C=O str.), 1563(C=N), 1437,1346(aromatic str.), 492(Co-N), 437(Co-O). ^1^H-NMR (DMSO-*d*_6_, 300 MHz, *δ*/ppm): 8.22-7.43 (14H, m, 2× naphthyl), 5.85–5.92 (2H, s, 2× NH), 4.15–4.08 (2H, s, olefinic), 3.83 (4H, m, 2× CH_2_), 2.73–2.55 (6H, m, 2× CH_3_ olefinic), 2.10–2.04 (6H, s, 2× CH_3_ azomethine); ^13^C-NMR (DMSO-*d*_6_, 75 MHz, *δ*/ppm): 181.61, 174.33, 168.34, 133.82, 131.71–124.51, 105.98, 95.10, 33.64, 19.72, 17.23. λ_max_ (nm): 330. *m*/*z*: 805

(ix) ((*E*)-*N*’-(1-(4-hydroxy-6-methyl-2-oxo-2H-pyran-3-yl)ethylidene)-2-(naphthalen-2-yl)acetohydrazide)2Cd.

White solid. Yield: 55%; m.p. >350 °C. Anal. Calcd. for C_40_H_34_N_4_CdO_8_ (*Mw* = 812.5 gmol^−1^). C, 59.23; H, 4.23; N, 6.91%. Found: C, 60.47; H, 4.28; N, 6.85%. FT-IR (KBr, cm^−1^): 3142 (N-H str.), 2962 (C-H str.), 1676 (C=O str.), 1624 (C=N), 1427, 1452 (aromatic str.), 482 (Co-N), 434 (Co-O). ^1^H-NMR (DMSO-*d*_6_, 300 MHz, x *δ*/ppm): 8.07–7.39 (14H, m, 2× naphthyl), 5.54 (2H, s, 2× NH), 4.12 (2H, s, olefinic), 3.35 (4H, m, 2× CH_2_), 2.50–2.49 (6H, m, 2× CH_3_ olefinic), 2.32–2.01 (6H, s, 2× CH_3_ azomethine); ^13^C NMR (DMSO-*d*_6_, 75 MHz, *δ*/ppm): 170.34, 159.91, 133.76–124.50, 108.18, 41.32-39.14, 20.63, 19.44. λ_max_ (nm): 360. *m*/*z*: 813

### 3.3. Matrix-Assisted Laser Desorption Ionization (MALDI) Protocol

The mass analysis of compounds was carried out by MALDI instrument (standard mixture under 6000 *m*/*z* Ultra Flex Analysis. UCLA, USA.), The matrix consisting of DHB (2,4-dihydroxybenzoic acid) in 100 µL of 50% Acetonitrile in water was prepared in a small sample vial and mixed well on the rotary mixer. MALDI steel plate containing 300 spots was first washed with water followed by washing with EtOH to get rid of any traces of residual impurities. A volume of 1 µL of the matrix was spread on every multiple circular spot then a non-calculated little quantity of ligand and metal complexes were added on them with the help of a pipette, and on these spots, another layer of the matrix was added to make a sandwich. It was air-dried for 3 min, the plate was inserted in the instrument, and data were recorded after taking multiple flashes on centroid spots to get maximum fragmented peaks by using low mass MALDI standard mixture under 6000 *m*/*z* Ultra Flex Analysis. The acquisition of data was in the form of a graph between relative abundance and *m*/*z*.

### 3.4. Micellar Interaction of Metal Complexes

Since the complexes were not completely soluble in pure water therefore their interaction was studied initially in a mixture of DMSO and distilled water (1:1). The slight change in the maximum absorbance of metal complexes was observed with redshifts, which indicated interaction and solubilization in micellar media. Both surfactants showed interaction to different extends. The comparative table of both surfactants’ interaction with complexes is presented in Table 4. 

#### 3.4.1. Preparation of Stock Solutions and Dilution Method

The stock solutions of all metal complexes (1 mM) were prepared in distilled de-ionized water and DMSO (1:1) and these primary solutions were used subsequently for the preparation of secondary solutions by dilution method with surfactant solutions for spectroscopic measurements. The secondary solutions were prepared by dissolving different amounts of surfactants from pre-micellar to post-micellar concentrations (2.5–6.5 mM for SS and 0.1–2.0 mM for CTAB). The stock solutions were diluted to hold the Lambert-Beer’s law.

#### 3.4.2. UV-Vis Spectroscopic Measurements

UV-vis absorbance values (simple and differential) were measured for the complexes at 298 K maintaining ±0.5K accuracy control in UV-vis spectrophotometer (Shimadzu, UV-1700, Kyoto, Japan). In the case of simple absorbance distilled water was taken as a reference standard while for differential spectroscopic analysis metal complex solution was taken in reference cell and the aqueous solutions of metal complexes in the presence of surfactants were taken in the sample cell. All the samples were homogenized and rested for overnight to attain equilibrium before measurements.

#### 3.4.3. Calculation of Partitioning and Binding Parameters

The partitioning and binding parameters were calculated from the spectroscopic data using the Equations (1)–(5) as given below.

(i) Partitioning coefficient (*K_x_*)

(1) $$\frac{1}{\Delta A}=\frac{1}{K_{C}\Delta A_{\infty }\left(C_{a}+C_{s}^{m_{{}^{\circ}}}\right)}+\frac{1}{\Delta A_{\infty }}$$

(2) $$K_{x}=K_{c}n_{w}$$

The differential absorbance (Δ*A*) helped to calculate binding constant *K_c_* which was then used to calculate portioning *K_x_* (Equations (1) and (2)).

(ii) Binding constant (*K_b_*)

(3) $$\frac{C_{S}C_{a}}{\Delta A}=\frac{C_{S}}{\Delta \epsilon l}+\frac{1}{K_{b}\Delta \varepsilon l}$$

(iii) Standard change in free energy of partition (Δ*G_p_*)

(4) $$\Delta G_{p}=-RTlnK_{x}$$

(iv) Standard change in free energy of partition (Δ*G_b_*)

(5) $$\Delta G_{b}=-RTlnK_{b}$$

In the above equations, *K_c_* represents partition constant; *C_a_* is the concentration of dye; Δ*A*_∞_ stands for differential absorbance at infinite dilution. The relationship $C_{s}^{m}$ = *C_s_* − CMC_o_; represents CMC of the surfactants in water as CMC_o_, while Cs is the concentration of surfactants. In Equation (2), *n_w_* shows the number of moles of H_2_O. The experimental values of these parameters are presented in the results section (Table 6).

#### 3.4.4. Electrical Conductivity Measurements

Electrical conductivity (EC) data were measured on Hanna Conductivity meter (HI 99301, Hanna Instruments Inc. Highland Industrial Park 584 Park East Drive Woonsocket, RI 02895 USA) in 0.01–199.9 mS range (±0.5% ± 2 accuracy control). The electrode calibration was ensured with potassium chloride over the desired micellar concentration range. The conductivities of all complexes in the presence surfactants were measured at 298 K temperature.

#### 3.4.5. Parameters Calculated by Conductivity Data

(i) Critical Micellar Concentration (CMC)

The concentration of surfactants at which at their process of micellization initiates is designated as critical micelle concentration (CMC). Micellization is a dynamic and complicated process so the measurement of exact and accurate micellar concentration is difficult because its value is estimated from a very narrow concentration range between pre-micellar and post-micellar regions. In micelle formation, the surfactants undergo a sudden change in their physicochemical properties, including conductivity.

(ii) The Degree of Dissociation (*β*)

The degree of dissociation was determined from the ratio of slopes of the conductivity–concentration plots in the pre-and post-micellar region (Equation (6)).

(6) $$\beta =\frac{S_{2\;\left(Post-micellar\;slope\right)}}{S_{1\;\left(Pre-micellar\;slope\right)}}$$

(iii) Free Energy of Micellization (Δ*G^°^_m_*)

(7) $$\Delta {G^{{}^{\circ}}}_{m}=\left(2-\beta \right)RT\ln X_{\mathrm{CMC}}$$

In Equation (7), *R* = Universal gas constant (8.314 J mol^−1^K^−1^); *T* = absolute temperature; *X*_CMC_ = critical mole fraction of surfactants at their respective CMCs. The experimental values of these parameters are presented in the results section (Table 4).

### 3.5. DNA Binding Activity Studies

UV-vis spectrophotometer (Shimadzu, UV-1700, Kyoto, Japan) was used to study the binding interaction of ligand, metal complexes, and the DNA under the range of 250–780 nm. The concentration of CT-DNA was determined spectrophotometrically by using an extinction coefficient of 6600 M^−1^ cm^−1^ at 260 nm. DNA interaction of free ligand and metal complexes were studied individually against CT-DNA having a 0.66 µM solution. The solution of CT-DNA was stirred for about 24 h by maintaining the temperature at 4 °C. The purity of CT-DNA free from protein was confirmed by an observed peak at 260 nm and by taking the ratio of absorbance on λ_max_ on 260 and 280 nm, which was 1.82. Concentrated stock solutions of the ligand and complexes were prepared in DMSO. The λ_max_ was determined by successive addition of 0.1 mL of DNA with the help of a micropipette in the sample solution.

### 3.6. Anti-Bacterial and Anti-Fungal Protocol

The 0.02 g of sample was dissolved in 1 mL of DMSO. Freshly prepared cultures of *E. coli*, *S. aureus*, Listeria, Pseudomonas, and Candida albicans were taken. Their suspensions were made by inoculating single colony of each organism into 10 mL of saline solution (0.9% NaCl). Muller Hinton Agar (MHA) was used as a growth medium for bacterial growth and Sabouraud Dextrose Agar was used for fungal growth. The culture was spread onto the Petri plates and digging of wells were carried out. Afterwards, those wells were being sealed by adding 0.5 mL of liquefied MHA into the wells and they were kept for a few minutes for drying. Then, the pre-prepared solution of compounds was poured (100 uL) into the wells against each microorganism. Thereafter, the Petri plates were kept overnight in an incubator. The plates were observed the very next day for results in terms of zones of inhibition.

### 3.7. MTT Based Cell Viability Assay

Cytotoxic activity of the ligand and its metal complexes was assessed in 96-well flat-bottomed microplates using the standard MTT (3-[4,5-dimethylthiazole-2-yl]-2, 5-diphenyl-tetrazolium bromide) colorimetric assay culturing HeLa cells, Cervical Cancer [25].

## 4. Conclusions

New ligands based on 2-(naphthalene-1-yl) acetic acid (NAA) hydrazone and metal complexes were successfully synthesized and characterized by FT-IR, ^1^H and ^13^C-NMRs, MALDI, and UV-visible spectroscopy. The synthesized complexes were evaluated for their solubilization in ionic surfactants viz. sodium stearate (sodium salt of stearic acid, SS) and Cetyltrimethylammonium bromide (CTAB). UV-Visible spectroscopy was employed to determine partitioning and binding parameters, whereas electrical conductivity measurements were employed to estimate critical micellar concentration (CMC), the extent of dissociation, and free energy of micellization. The complexes showed greater solubilization on the cationic micelles of CTAB as compared to SS owing to the electrostatic interactions of positively charged head group of CTAB and the negatively charged hydroxyl groups in the complexes. The experimental results showed an increase in the CMC values, and complexes interacted differently with both ionic surfactants. Finally, the electrostatic forces together with the nature of metal decided their positions in corresponding micellar systems. SS-DNA interaction studies with ligand as well as metal complexes individually displayed promising results by representing good values of binding constants and Gibbs free energy. The maximum binding was observed for the Cu(II) complex. All the complexes exhibit intercalation. Furthermore, anti-bacterial and anti-fungal activity results showed that only Cu(II) and Cd(II) complexes were active approximately against all strains. The HeLa cervical cancer cell lines result represented that Cu(II) and Pd(II) complexes are extremely active compared to the standard dose Doxorubicin. The performance and application of these drugs in various fields regarding their activity could be a positive turn in pharmaceuticals and from an industrial point of view.

## Figures and Tables

**Table 1:** Infra-Red absorption frequency of compounds.

Compounds	Infra-Red Bands (Stretching’s in cm^−1^)						
	υ(N-H)	υ(O-H)	υ(C=O)	υ(C=N)	υ(arom.)	υ(M-O)	υ(M-N)
Ligand	3275(w)	2997(w)	1702(s)	1654(s)	1511,1466	-	-
Co(L)2	-	3142(w)	1693(s)	1619(s)	1449–1428	568(m)	489(m)
Ni(L)2	3437(w)	-	1676(s)	1619(s)	1455–1428	570(m)	440(m)
Cu(L)2	-	3208(w)	1680(s)	1654(s)	1459–1428	538(m)	480(m)
Zn(L)2	3147(w)	-	1692(s)	1625(s)	1456–1442	527(m)	488(m)
Pd(L)2	3310(w)	-	1689(s)	1563(s)	1437–1425	492(m)	437(m)
Cd(L)2	3142(w)	-	1676(s)	1624(s)	1452–1427	434(m)	482(m)

**Table 2:** Partitioning and binding parameters of M(II) complexes in micellar media.

Surfactant	Complex	*K_c_* × 10^3^ (dm^3^/mol)	*K_x_* × 10^3^ (dm^3^/mol)	*K_b_* (dm^3^/mol)	Δ*G_p_* (kJ/mol)	Δ*G_b_* (kJ/mol)
	Co(II)	3.54	197	567	−30.2	−15.71
	Ni(II)	1.76	97.6	225	−28.46	−13.41
SS	Cu(II)	1.81	100	117	−28.53	−11.79
	Zn(II)	2.36	131	300	−29.19	−14.13
	Pd(II)	7.96	442	125	−32.2	−15.31
	Cd(II)	6.28	349	700	−31.61	−16.23
	Co(II)	1.52	84.2	2300	−28.09	−19.17
	Ni(II)	22.8	1270	16000	−34.81	−23.98
CTAB	Cu(II)	6.6	367	56700	−31.74	−21.41
	Zn(II)	1.83	102	1600	−28.56	−18.27
	Pd(II)	7.13	396	617	−31.93	−15.91
	Cd(II)	5.81	323	5000	−31.42	−21.1

**Table 3:** Micellar attributes of the metal complex in surfactants at 298 K.

Complex		SS		CTAB		
	CMC (mM)	Δ*G^°^_m_* (kJ mol^−1^)	*β*	CMC (mM)	Δ*G^°^_m_* (kJ mol^−1^)	*β*
Co(II)	4.48	−40.31	0.195	1.04	−42.56	0.276
Ni(II)	4.73	−41.89	0.274	1.11	−46.21	0.422
Cu(II)	4.28	−34.75	0.519	1.2	−45.41	0.294
Zn(II)	4.59	−37.30	0.399	1.24	−40.18	0.485
Pd(II)	4.85	−39.19	0.533	1.26	−44.58	0.229
Cd(II)	4.26	−34.44	0.307	0.99	−47.99	0.317

**Table 4:** Anti-bacterial activity of the ligand and its M(II) complexes expressed as the diameter (mm) of the inhibition zone.

Tested Compound	*E. coli*	Pseudomonas	*S. aureus*	Listeria	*C. albicans*
Ligand	-	5	-	-	-
Co(II) complex	-	5	-	-	-
Ni(II) complex	-	-	-	-	-
Cu(II) complex	10	5	10	10	-
Zn(II) complex	-	-	-	-	-
Pd(II) complex	-	-	-	-	5
Cd(II) complex	9	-	12	15	9

- inactive.

**Table 5:** Anti-cancer studies against Hela cancer cell lines.

Tested Compound	Conc. (mg/mL)	% Inhibition
Ligand	30 µM	26
Co(II) complex	30 µM	28
Ni(II) complex	30 µM	41
Cu(II) complex	30 µM	87
Zn(II) complex	30 µM	28
Pd(II) complex	30 µM	97
Cd(II) complex	30 µM	31
Doxorubicin	30 µM	95

**Table 6:** Shift in maximum absorbance of M(II) complexes in aqueous and micellar media.

Complex	λ_max_ (nm)		
	DMSO:H_2_O (1:1)	SS System	CTAB System
Co(II)	327	332	341
Ni(II)	351	357	368
Cu(II)	347	352	365
Zn(II)	333	339	348
Pd(II)	339	343	355
Cd(II)	373	378	387

1 mM of each metal complex was used to prepare stock solutions.

## Data Availability

Not applicable.

## Supplementary Materials

The comparative UV-vis analysis of ligand and metal complexes is given in Figure S1. The DNA binding spectra are given in Figures S2–S7. Selected NMR and FT-IR and MALDI spectra of ligands and complexes are reported in Figures S7–S28, while selected electronic spectral analysis of compounds is given in Table S1.

## Sample Availability

Not available.
