# Supplementary material for: Synthesis of New Naphthyl Aceto Hydrazone-Based Metal Complexes: Micellar Interactions, DNA Binding, Antimicrobial, and Cancer Inhibition Studies
Source: Molecules. 2021 Feb 17;26(4):1044. doi: 10.3390/molecules26041044 (PMC7923181; doi:10.3390/molecules26041044)
Supplement: Supplementary file 1 [file molecules-26-01044-s001.pdf]

Supplementary Material

Synthesis of New Naphthyl Aceto Hydrazone-Based Metal Complexes: Micellar Interactions, DNA Binding, Antimicrobial, and Cancer Inhibition Studies

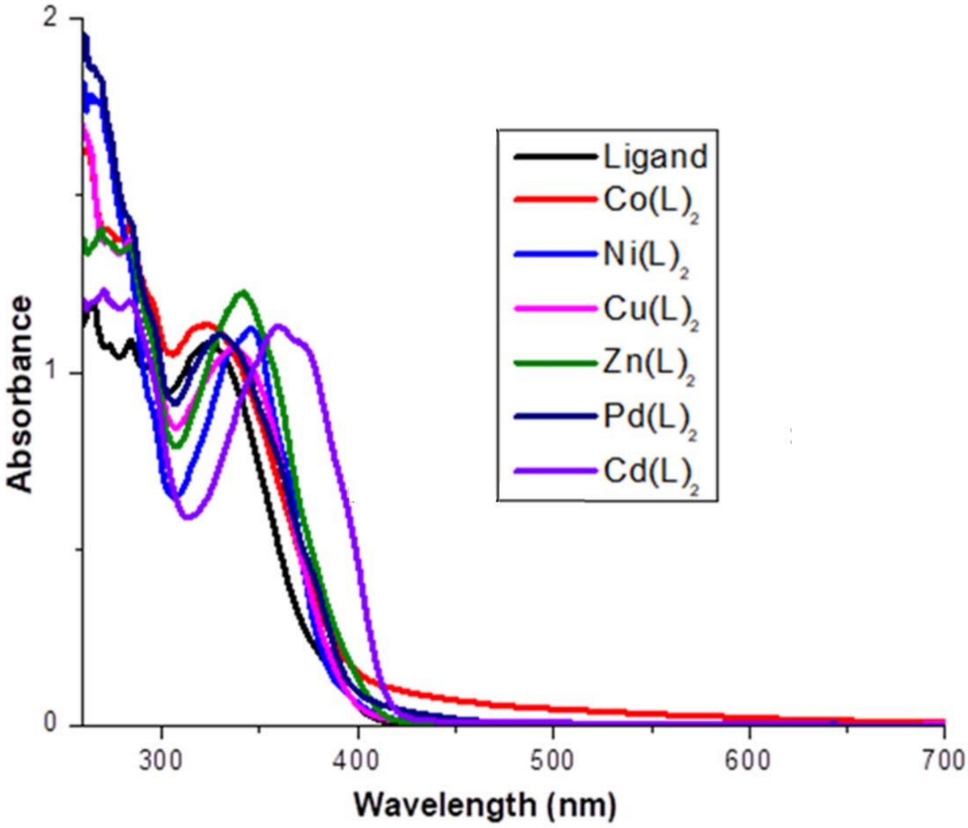

Figure S1. Comparative illustration for UV-vis absorption bands of ligand and metal complexes.

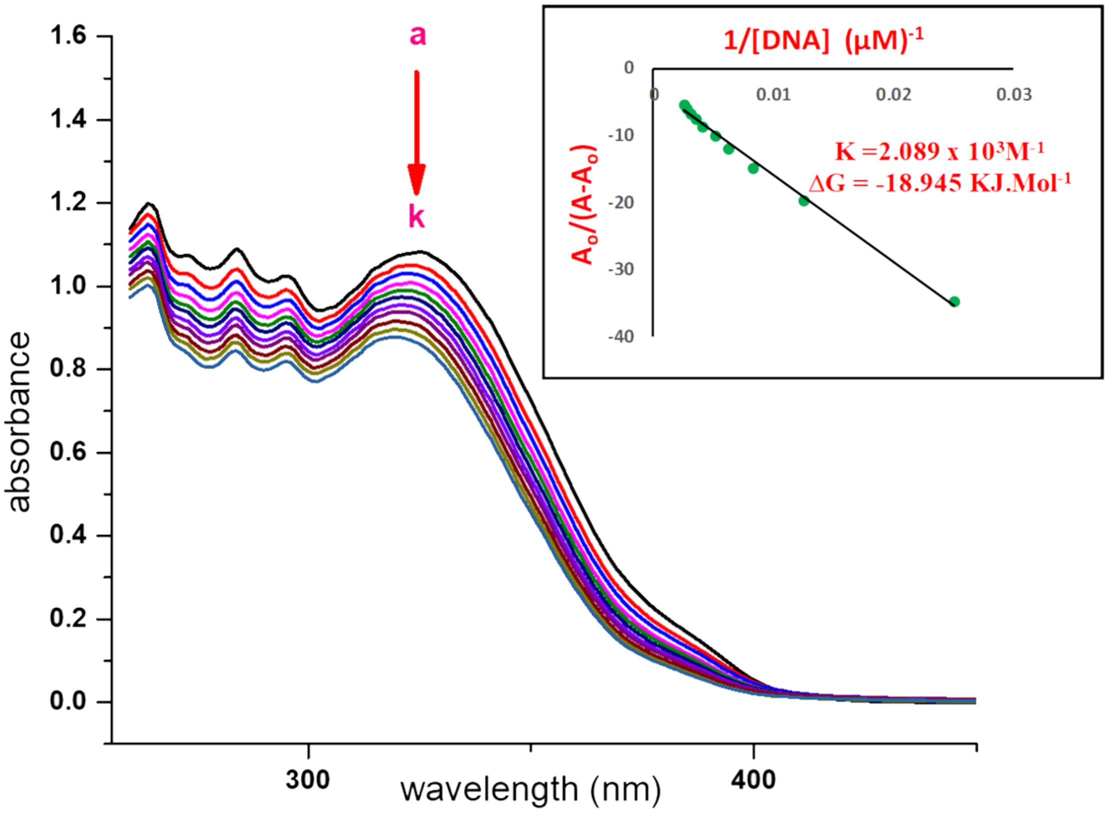

Figure S2. DNA interactions with ligand.

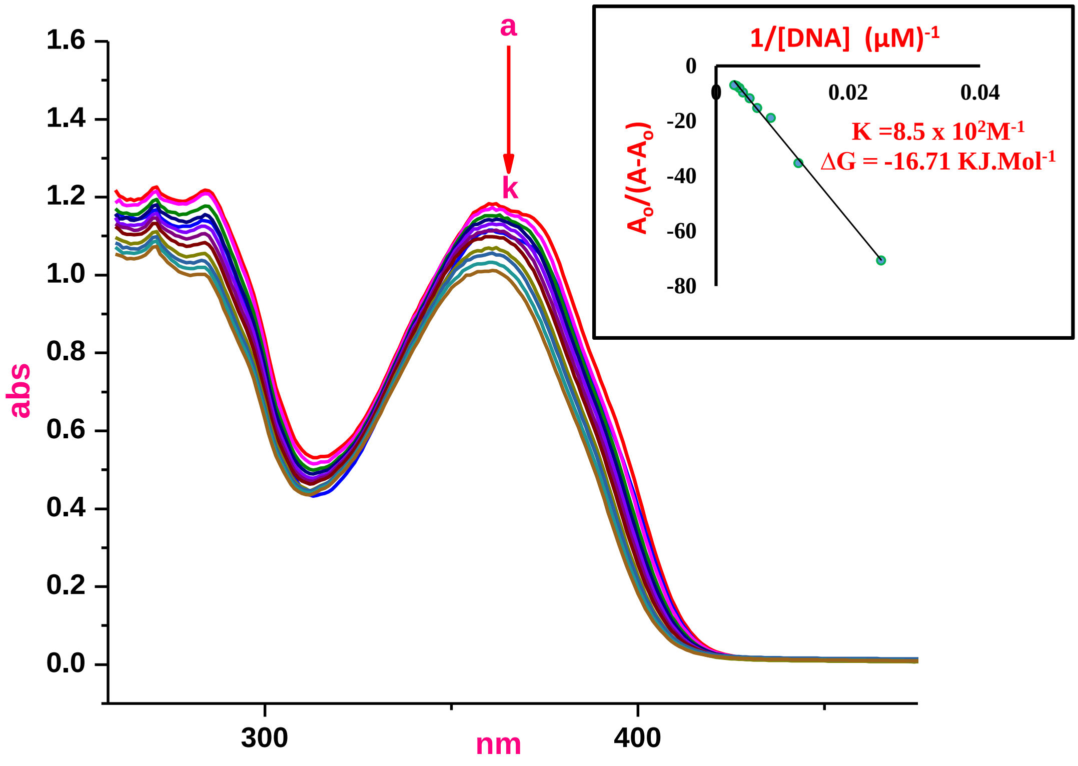

Figure S3. DNA interaction with Cd-complex.

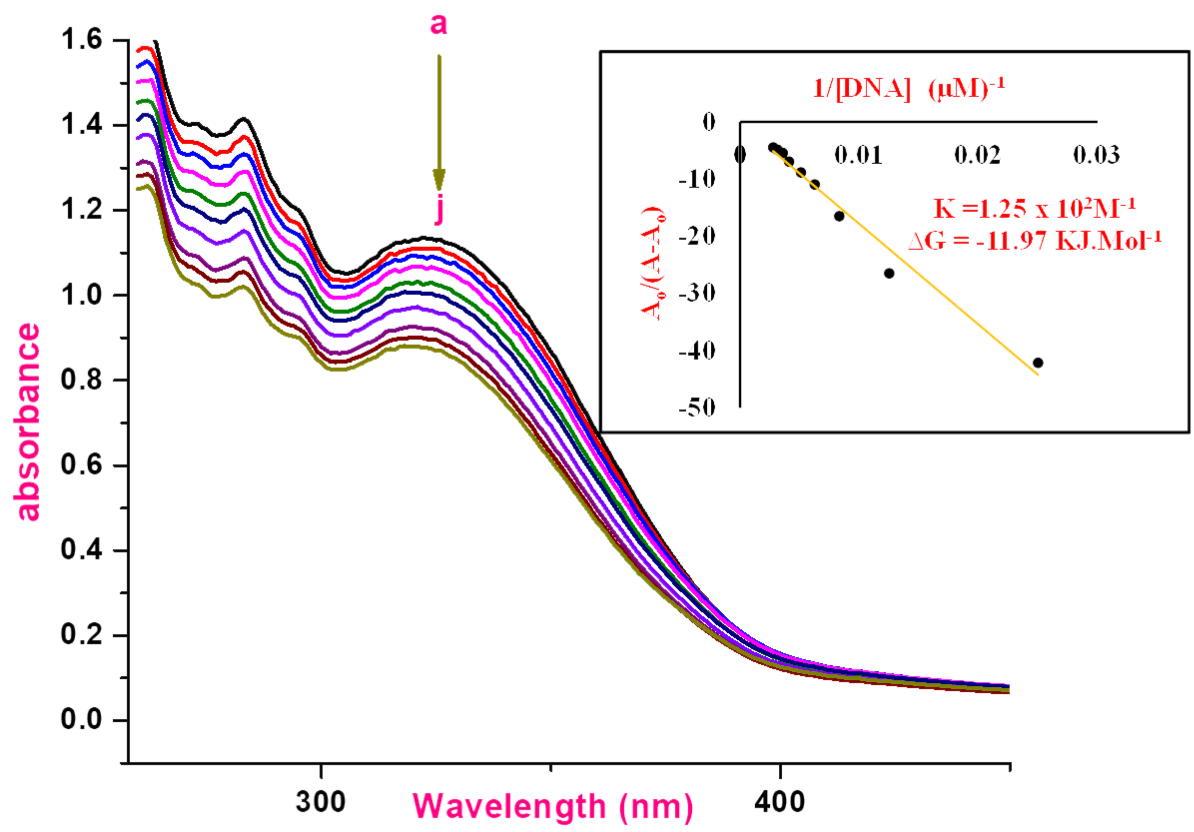

Figure S4. DNA interactions with Co-complex.

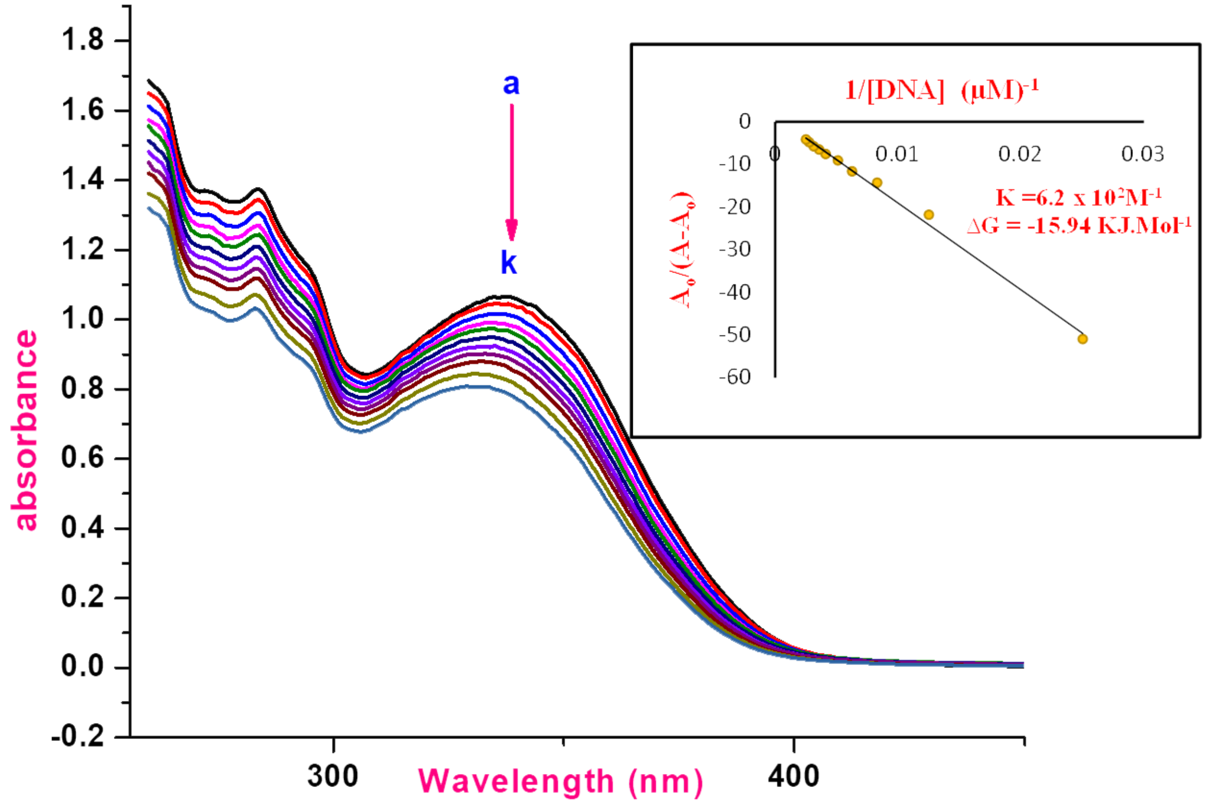

Figure S5. DNA interactions with Cu-complex.

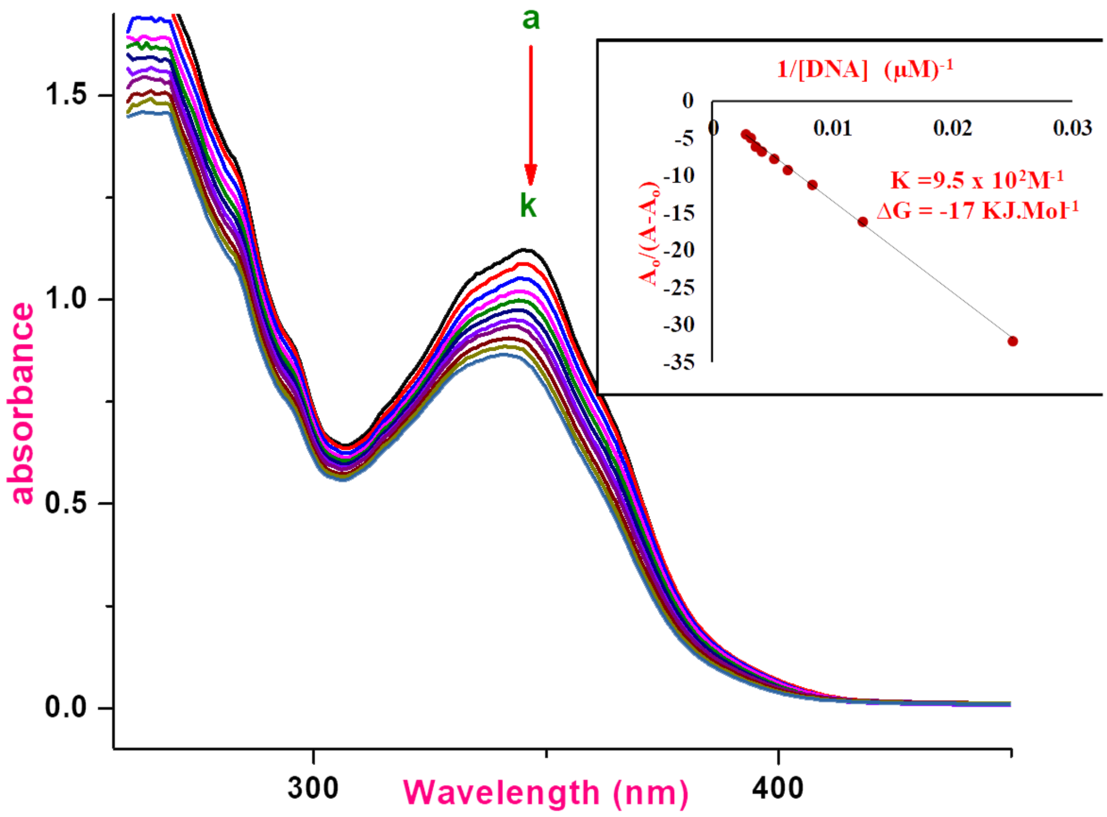

Figure S6. DNA interactions with Ni-complex.

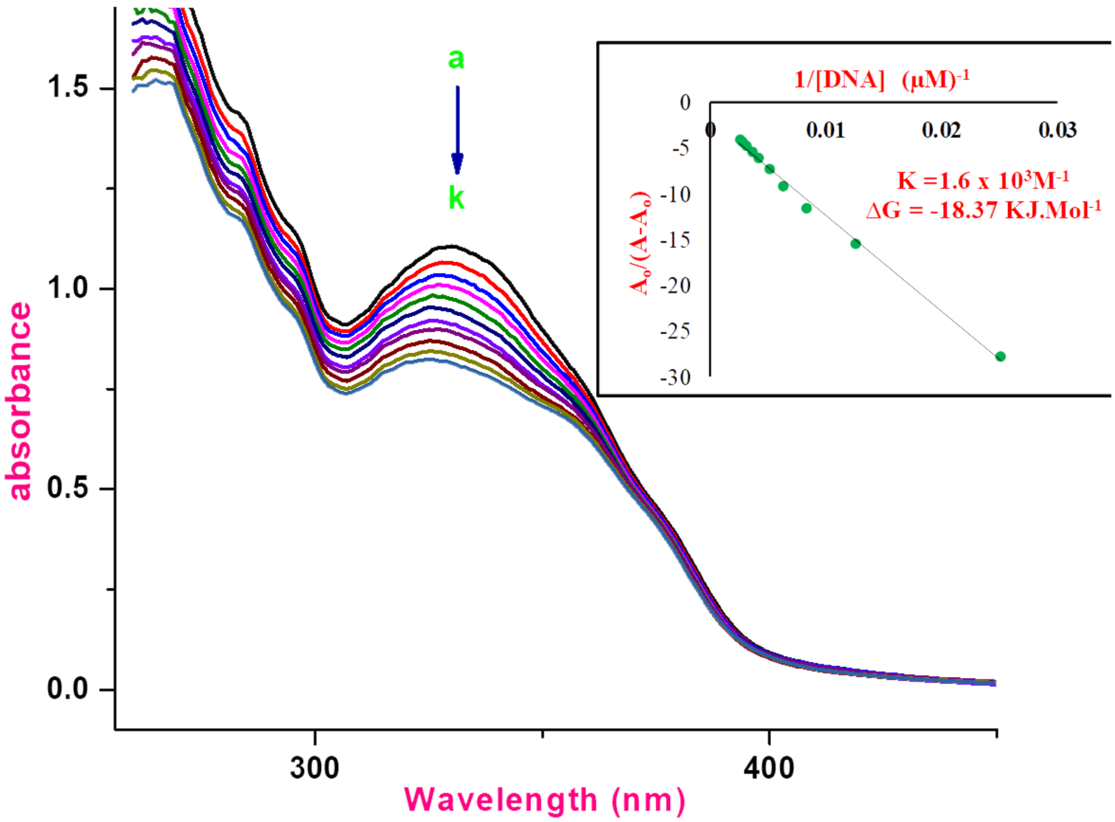

Figure S7. DNA interactions with Pd-complex.

SPECTRAS OF LIGANDS AND METAL COMPLEXES

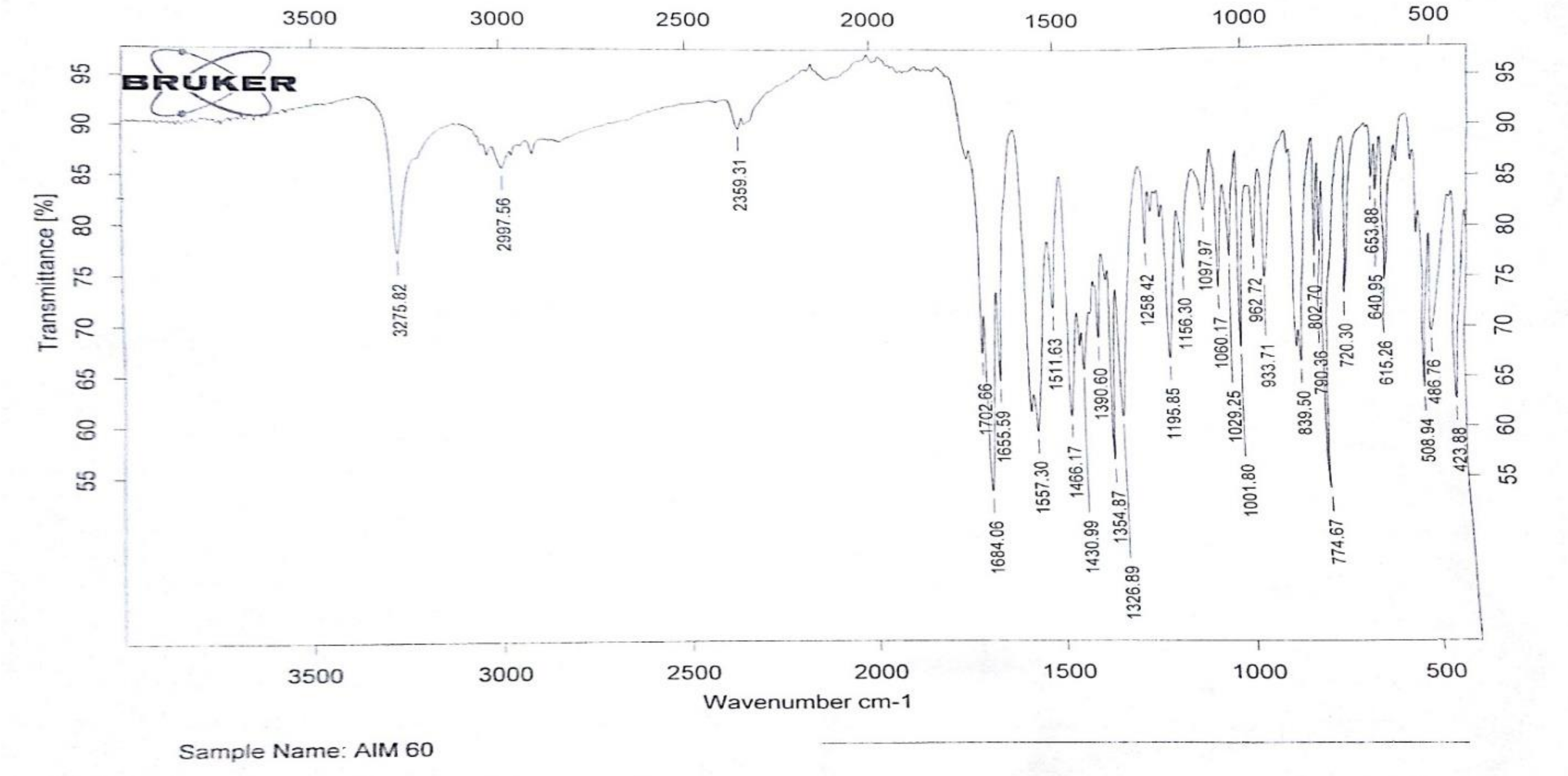

Figure S8. FTIR spectrum of Ligand.

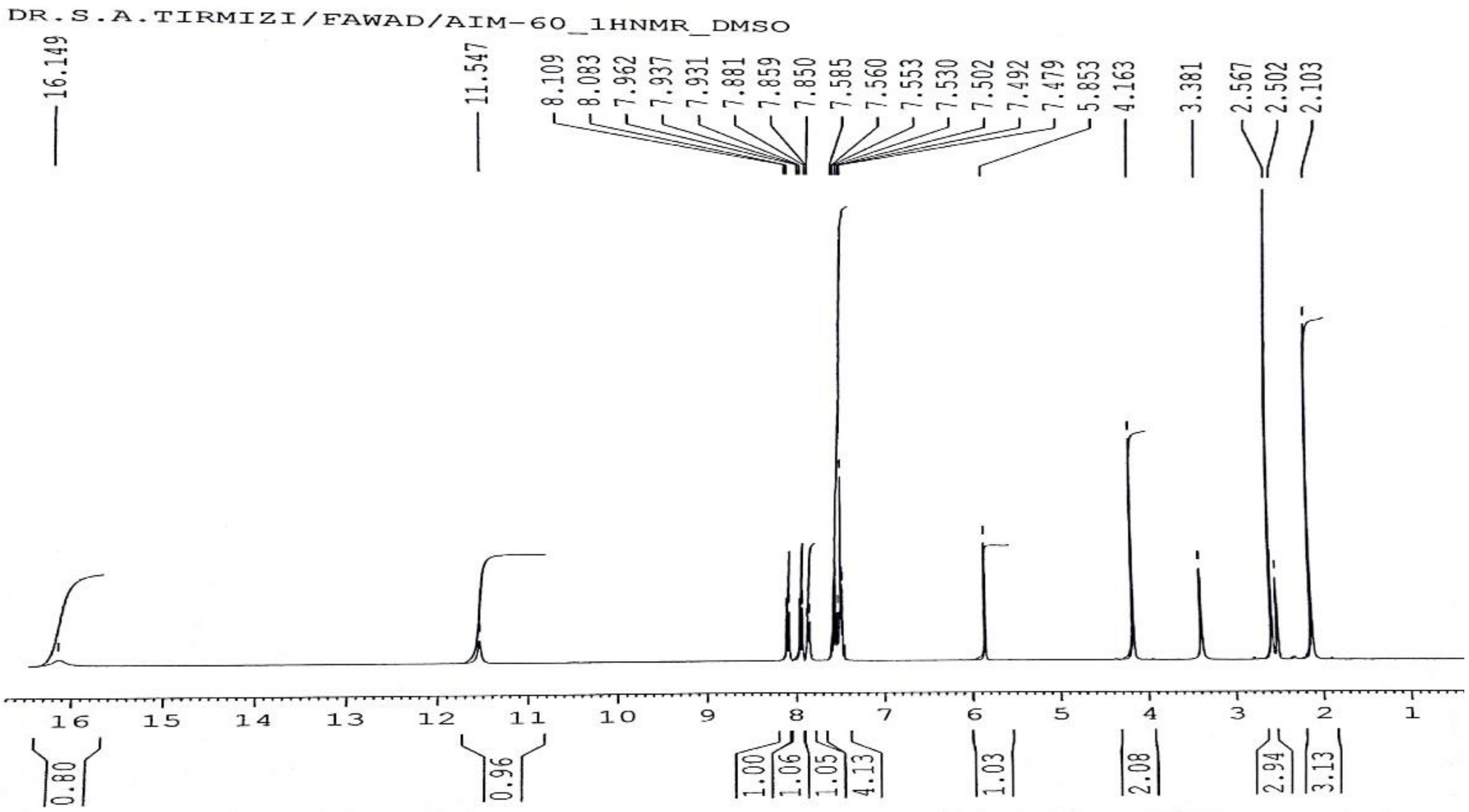

Figure S9. ^{1}H-NMR spectrum of Ligand.

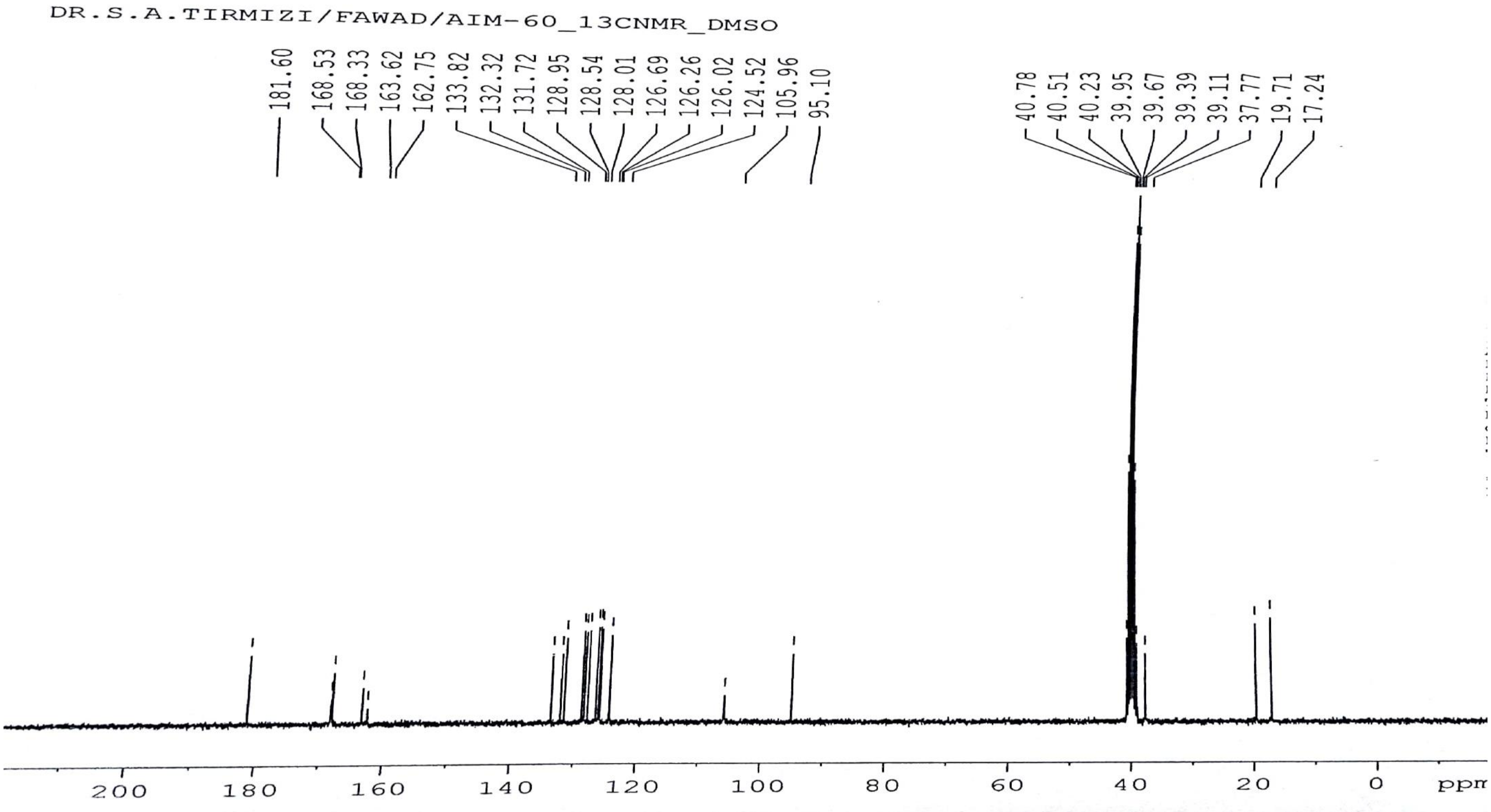

Figure S10. ^{13}C-NMR spectrum of Ligand.

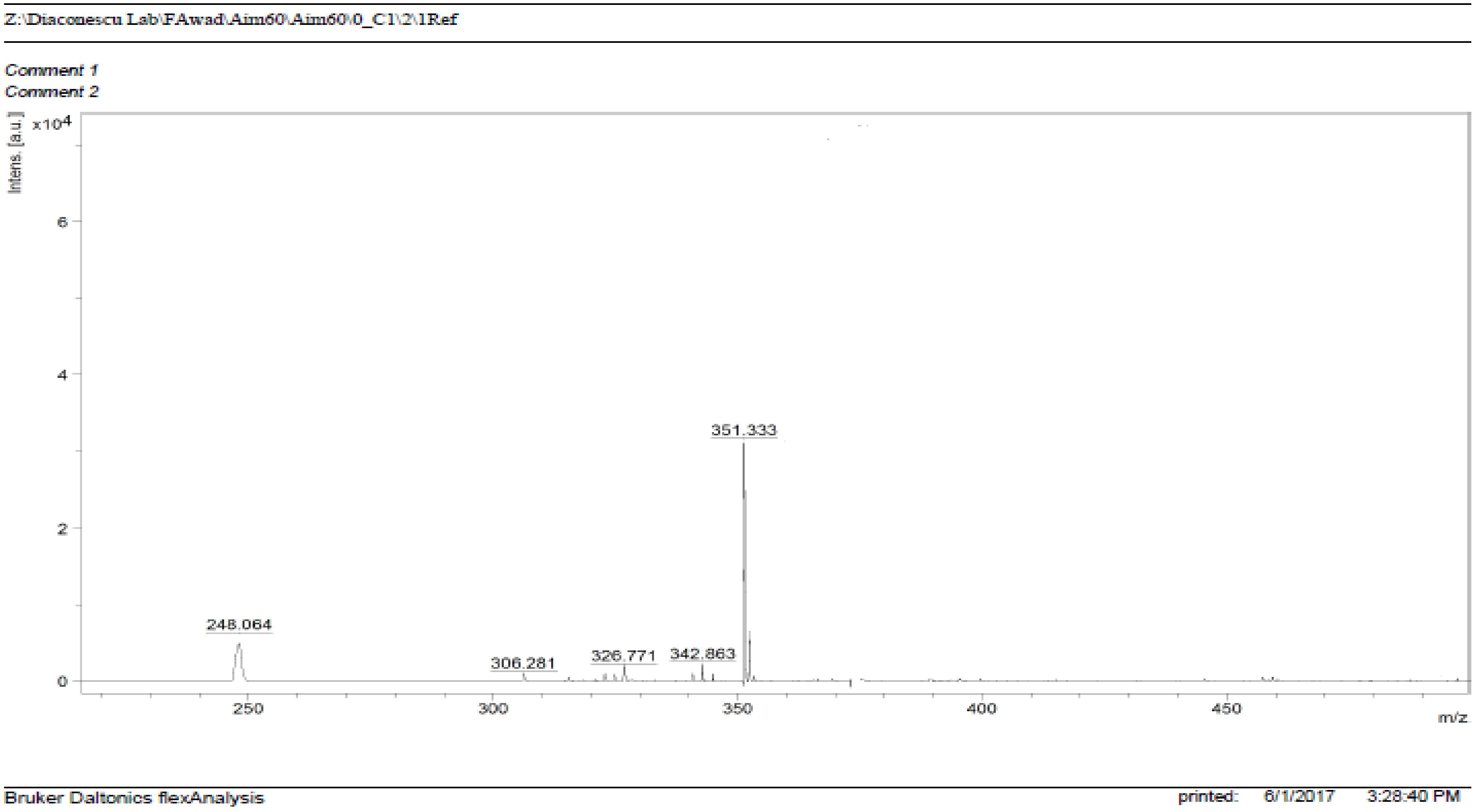

Figure S11. MALDI spectrum of Ligand.

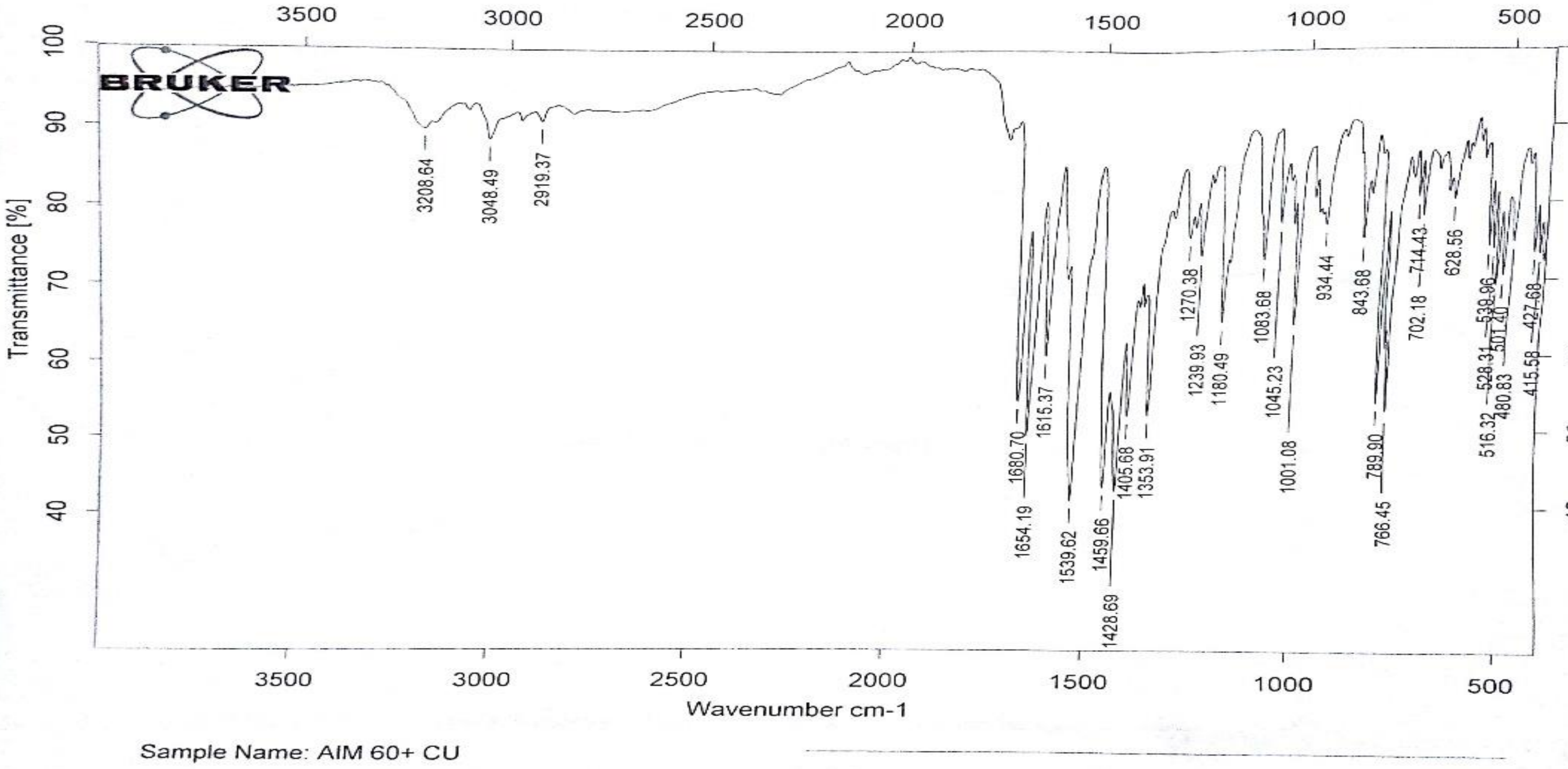

Figure S12. FTIR spectrum of Cu(II) Complex.

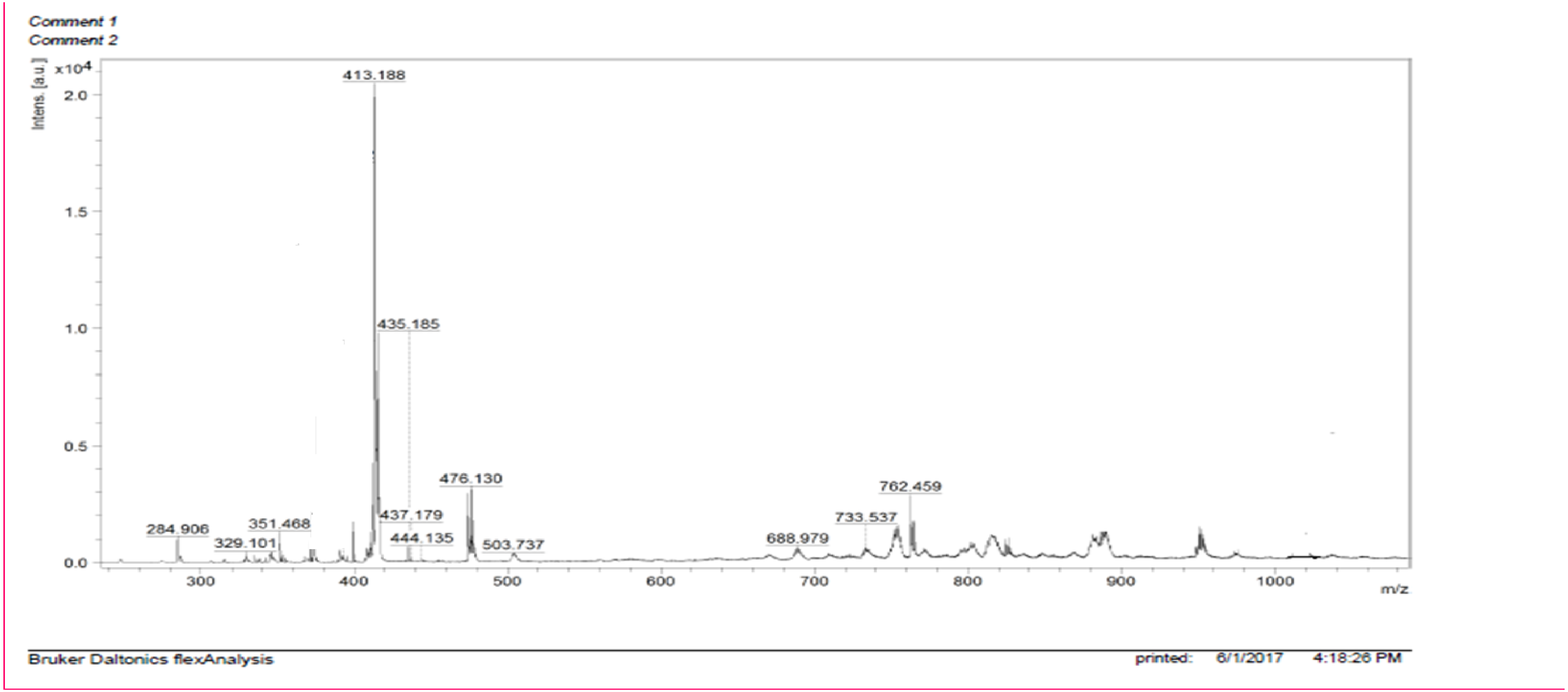

Figure S13. MALDI spectrum of Cu(II) Complex.

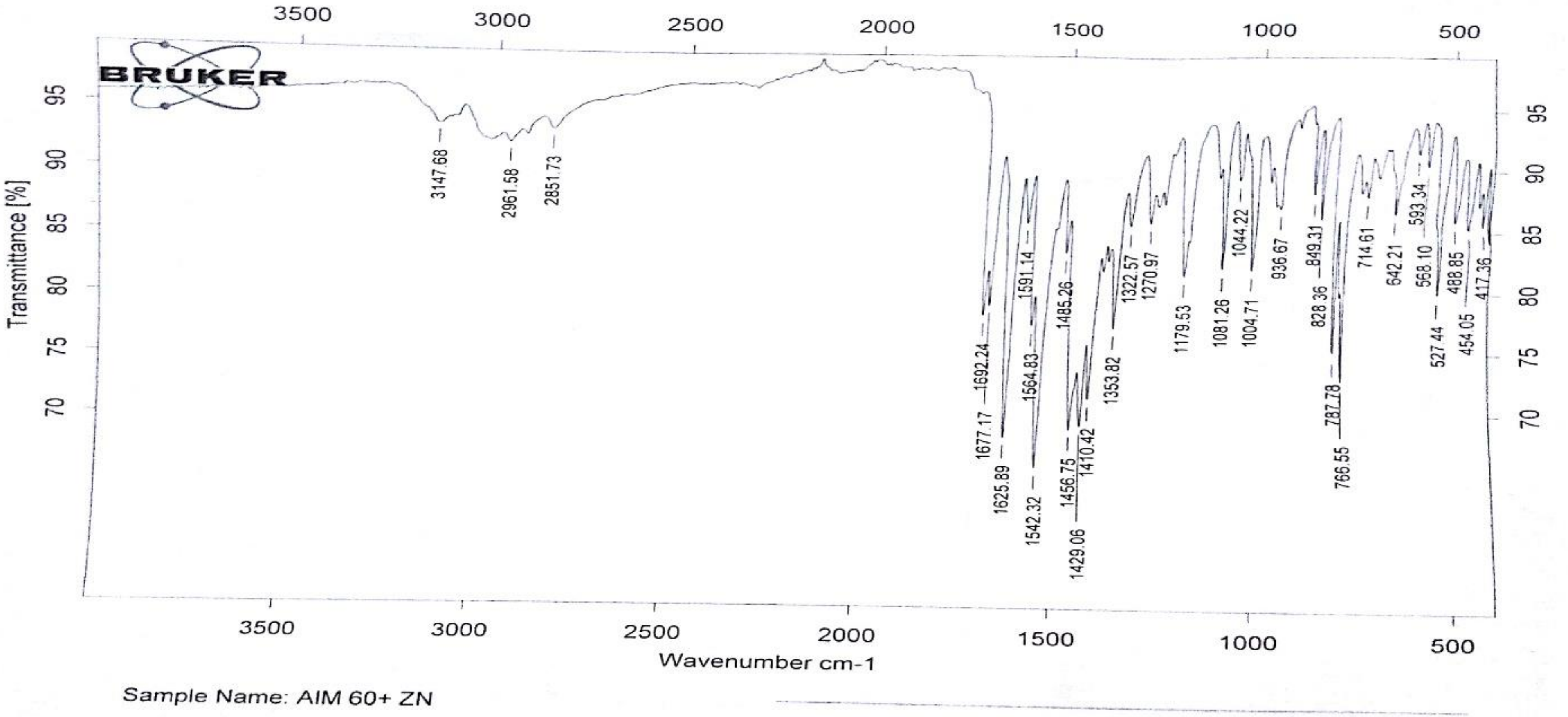

Figure S14. FTIR spectrum of Zn(II) Complex.

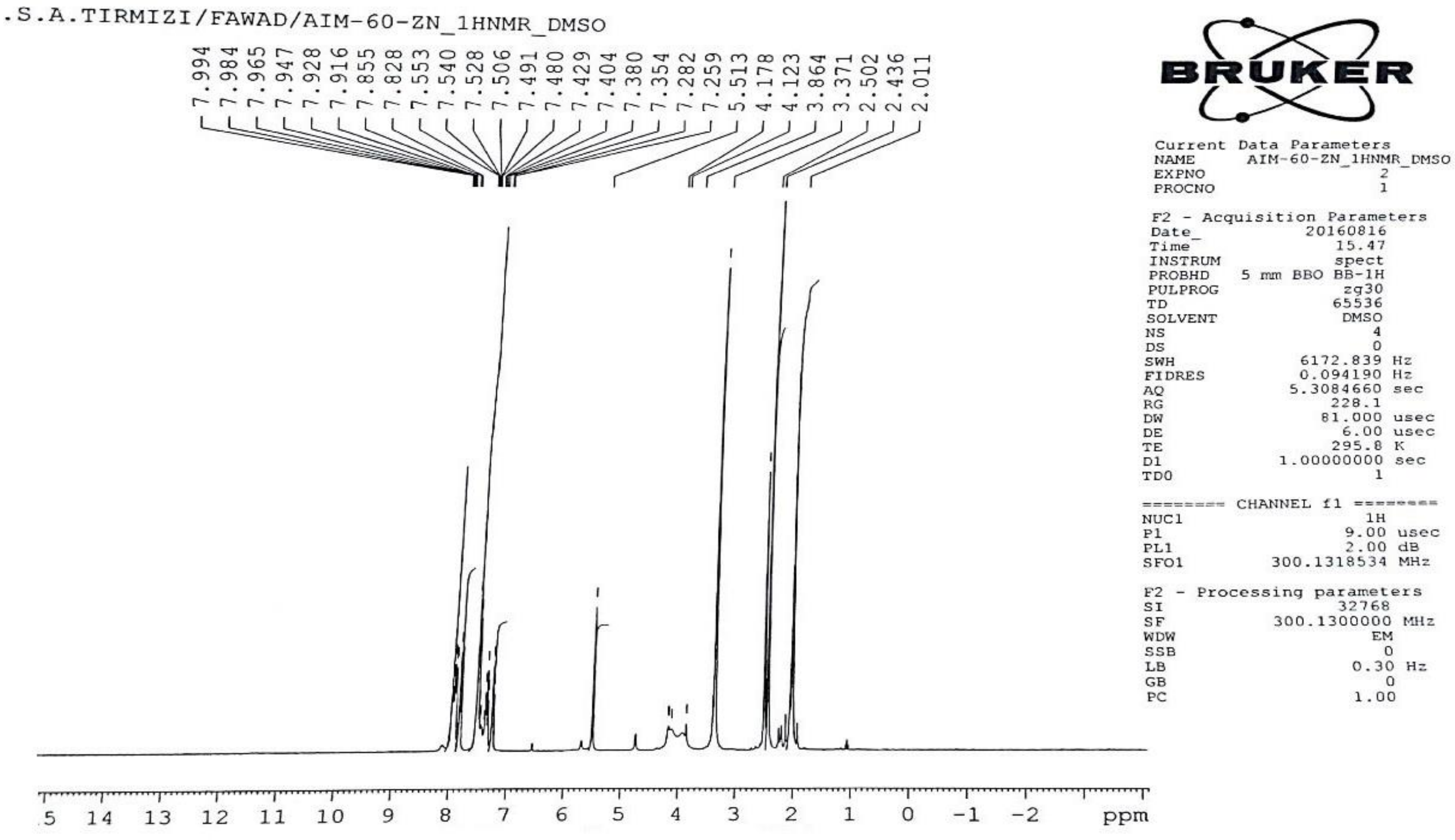

Figure S15. ^{1}H-NMR spectrum of Zn(II) Complex.

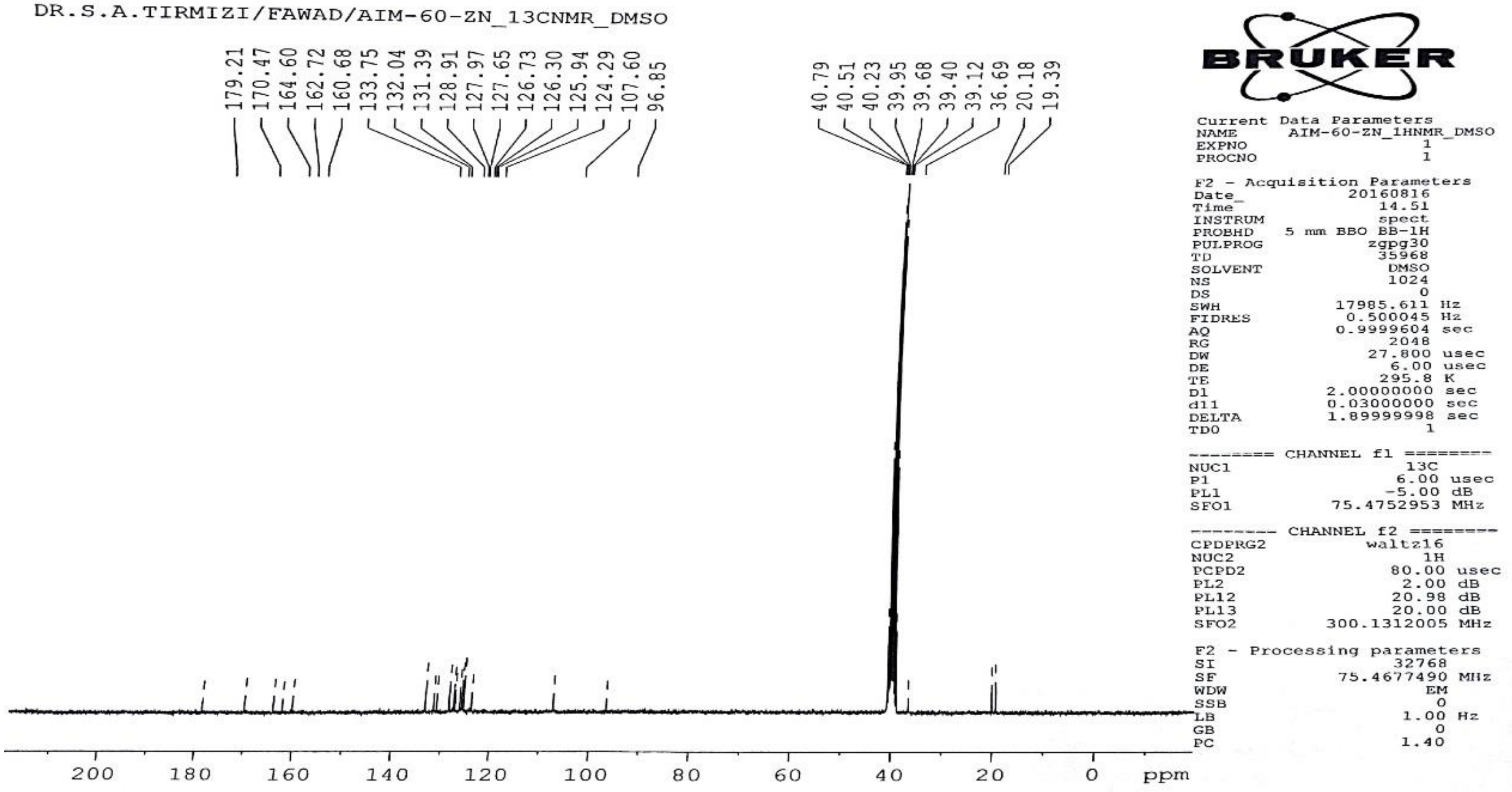

Figure S16. ^{13}C-NMR spectrum of Zn(II) Complex.

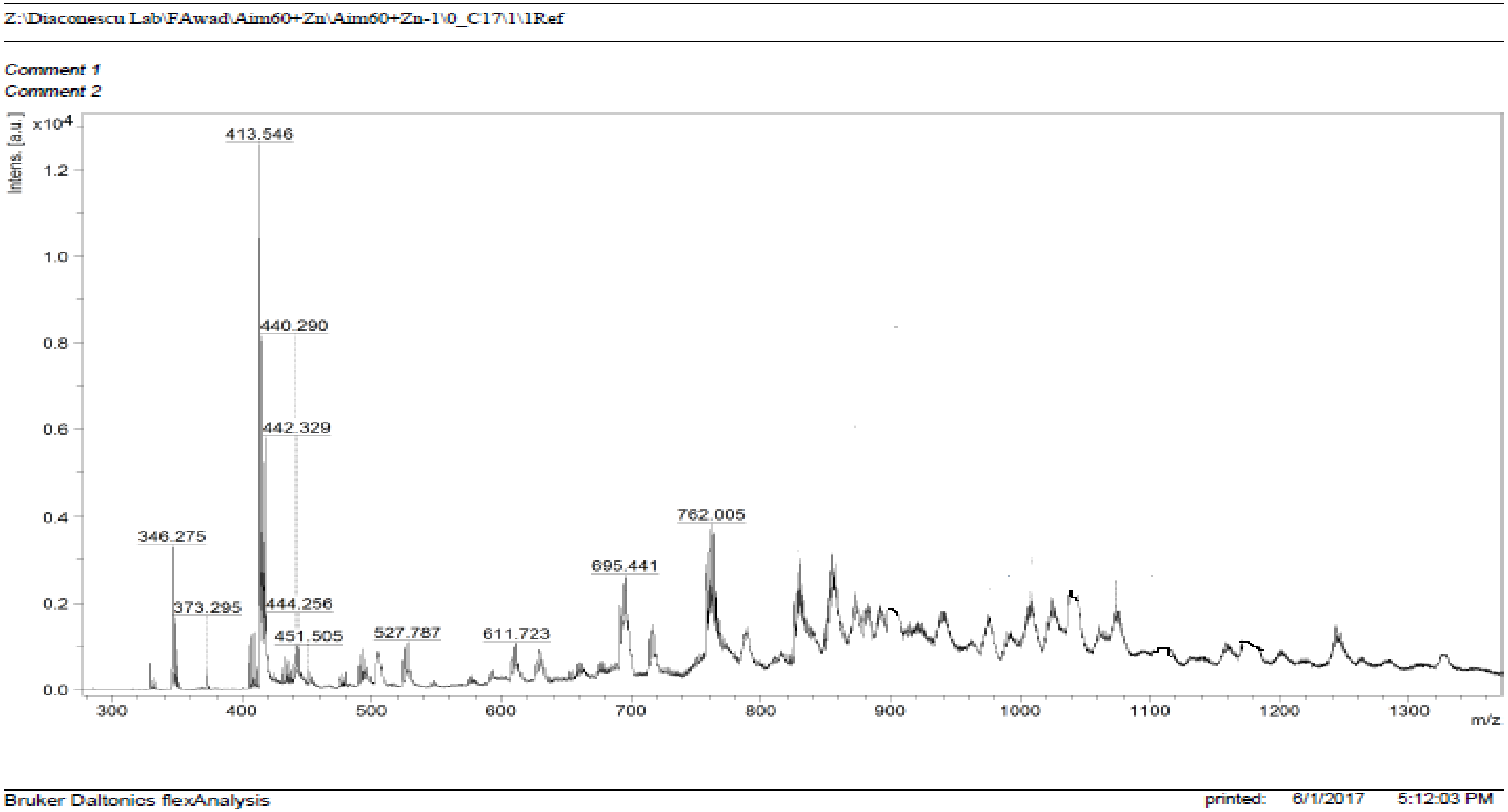

Figure S17. MALDI spectrum of Zn(II) Complex.

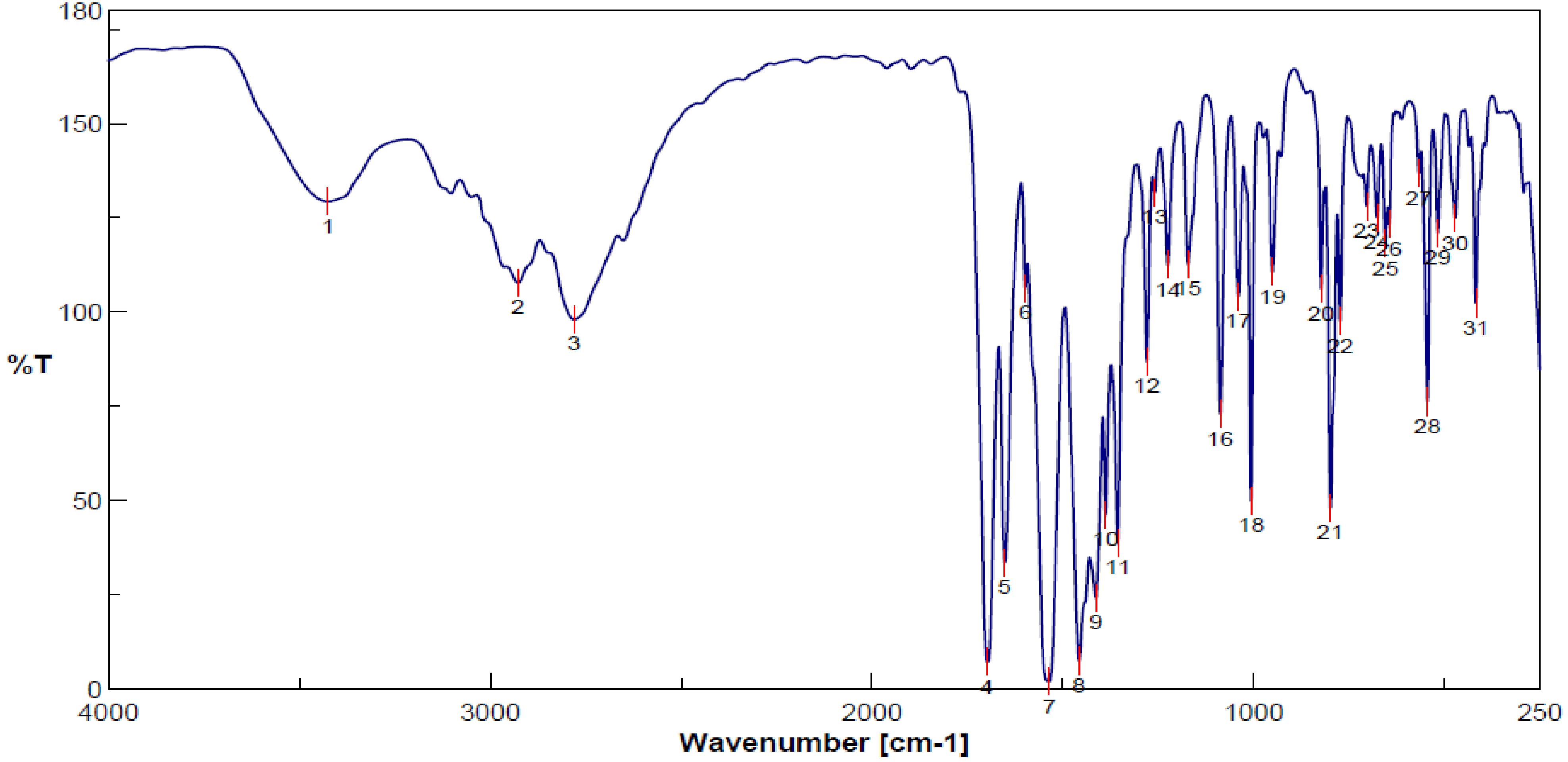

Figure S18. FTIR spectrum of Ni(II) Complex.

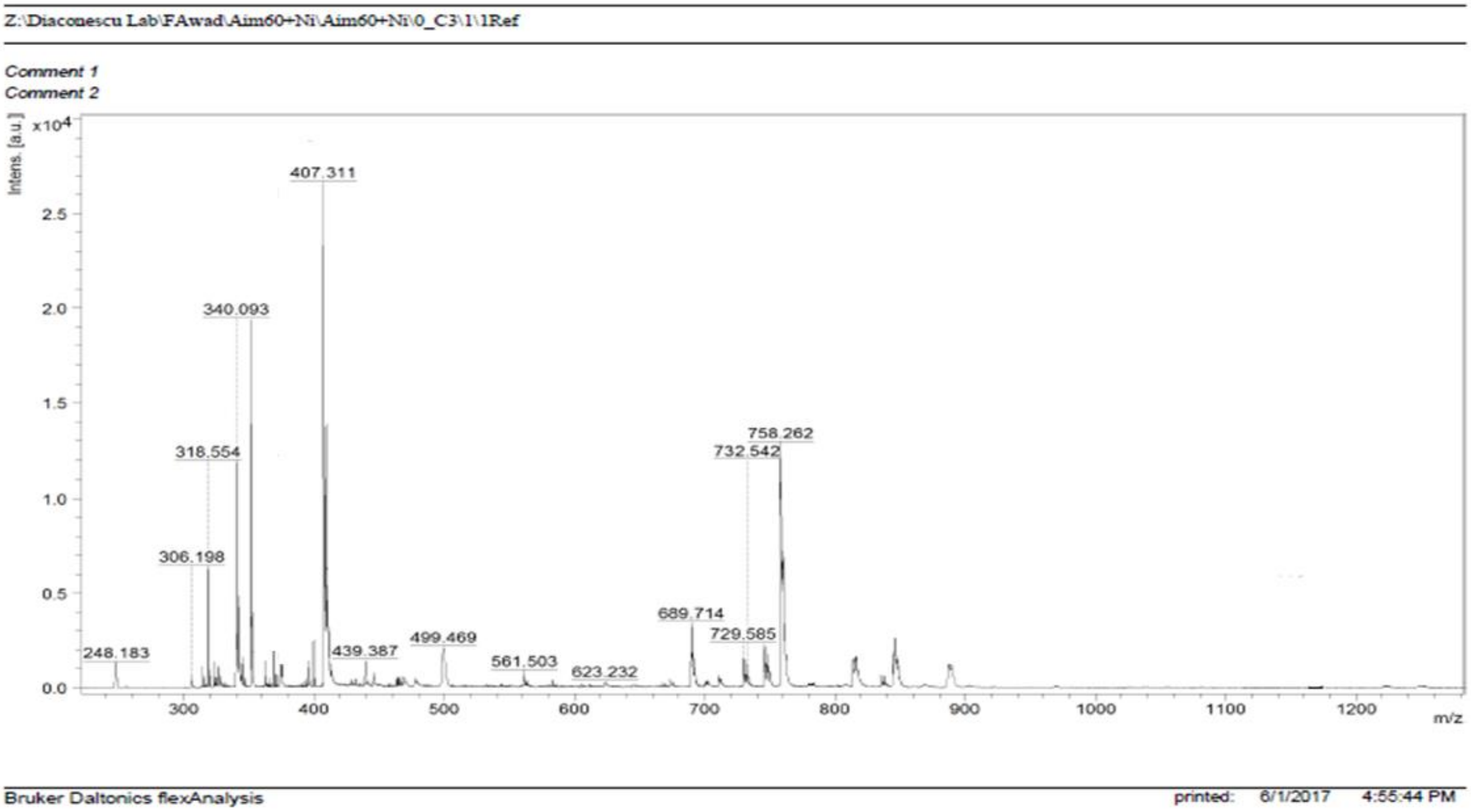

Figure S19. MALDI spectrum of Ni(II) Complex.

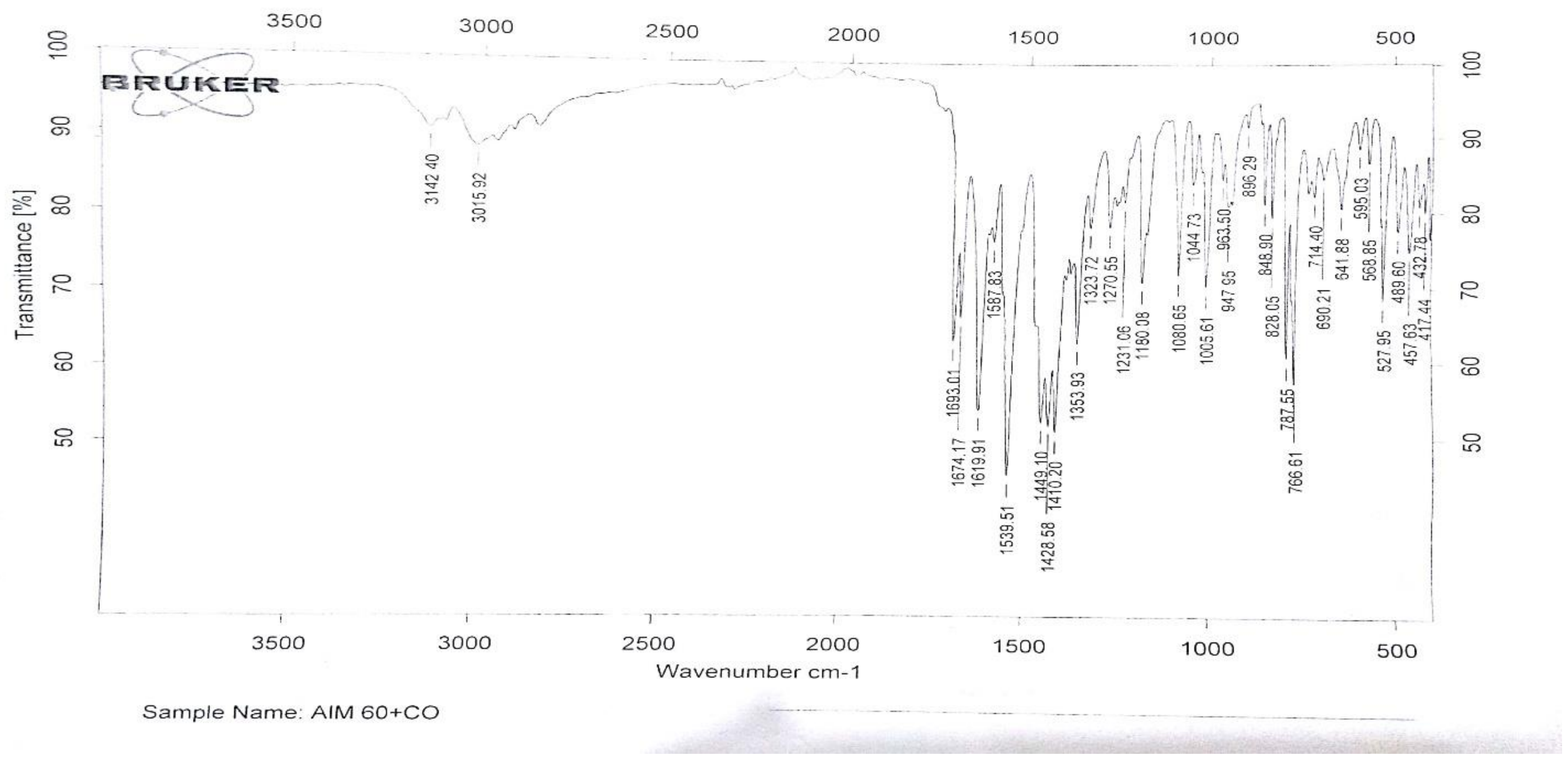

Figure S20. FT- IR spectrum of Co(II) Complex.

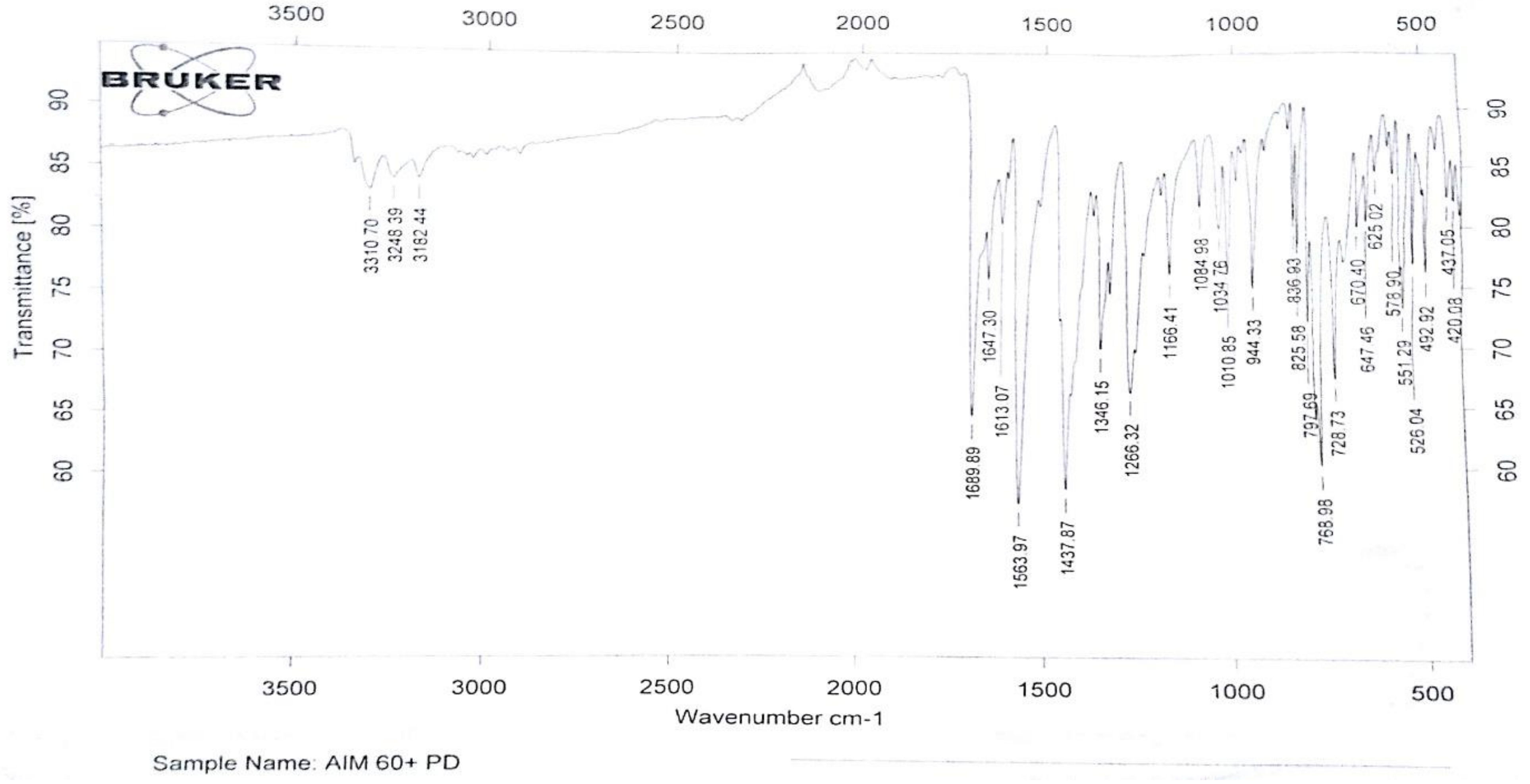

Figure S21. FTIR spectrum of Pd(II) Complex.

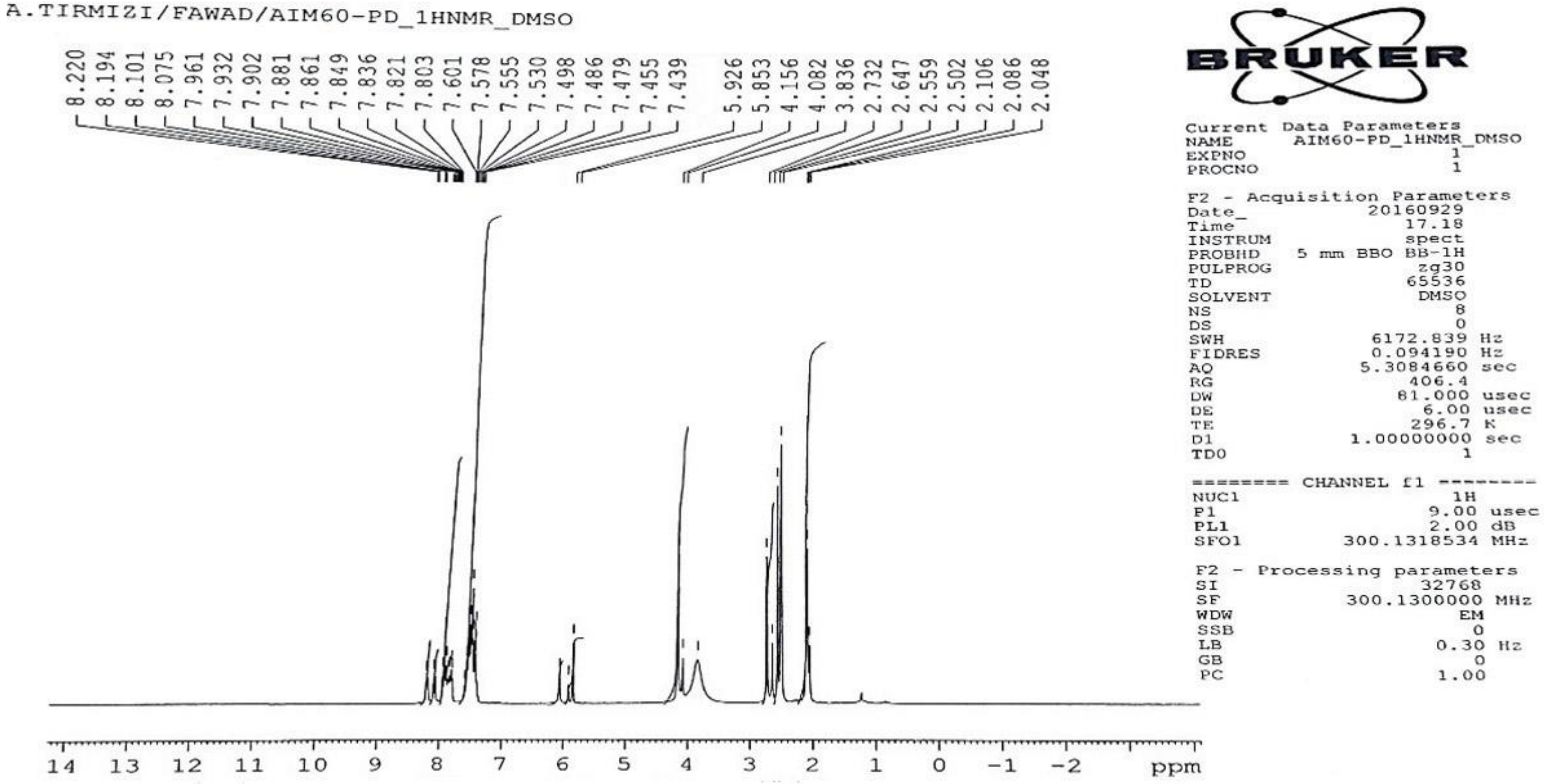

Figure S22. ^{1}H-NMR spectrum of Pd(II) Complex.

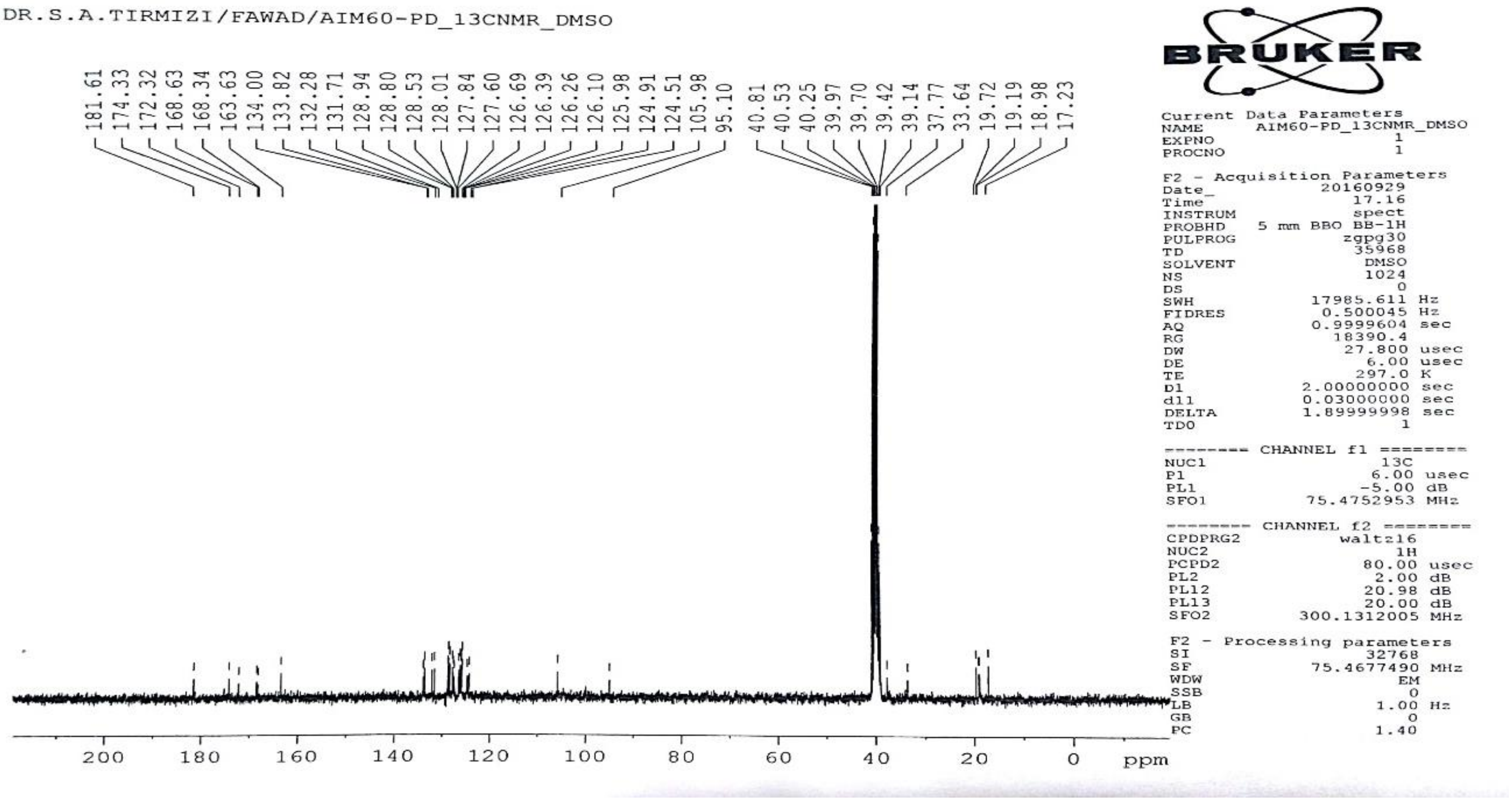

Figure S23. ^{13}C-NMR spectrum of Pd(II) Complex.

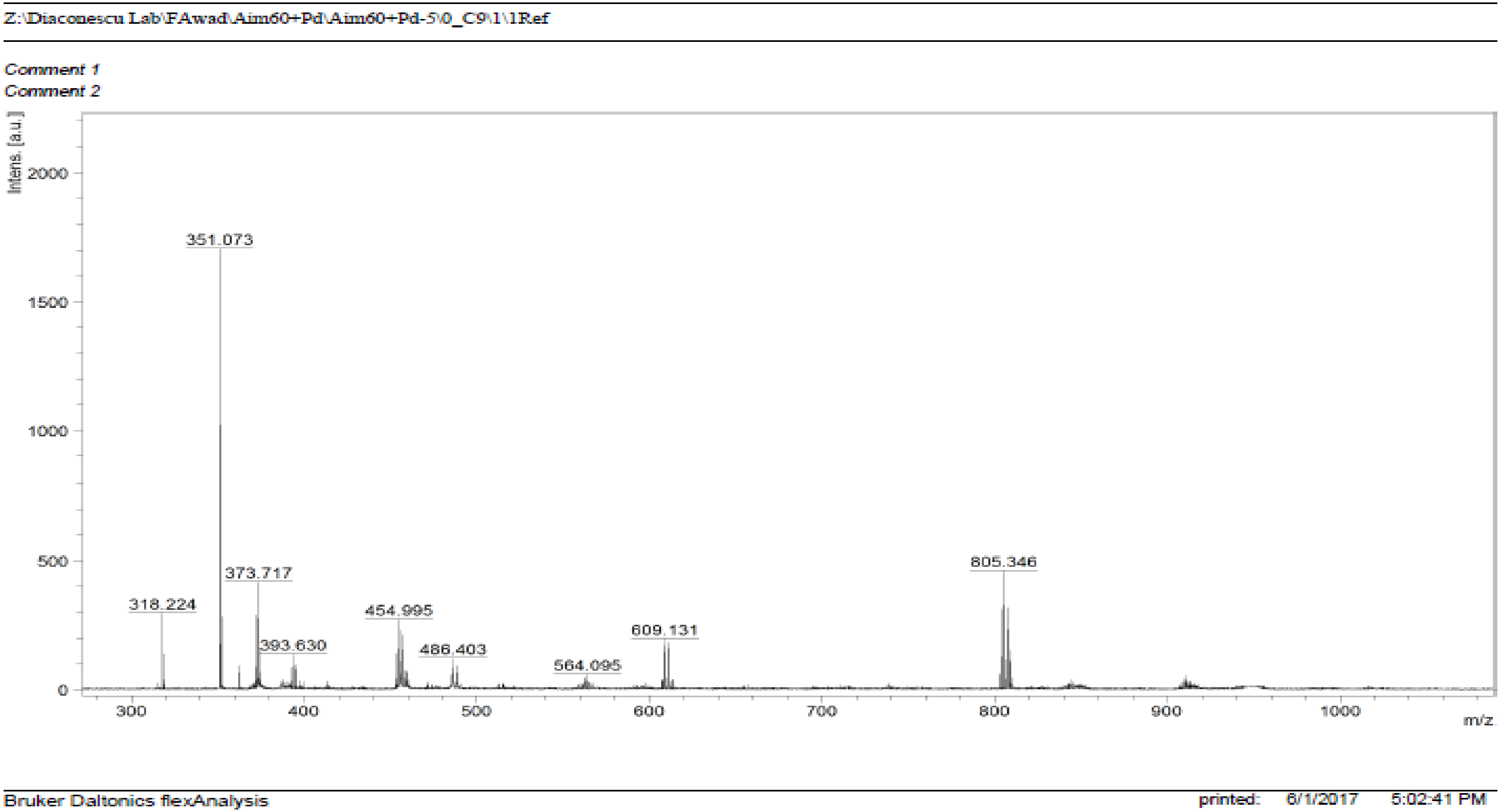

Figure S24. MALDI spectrum of Pd(II) Complex.

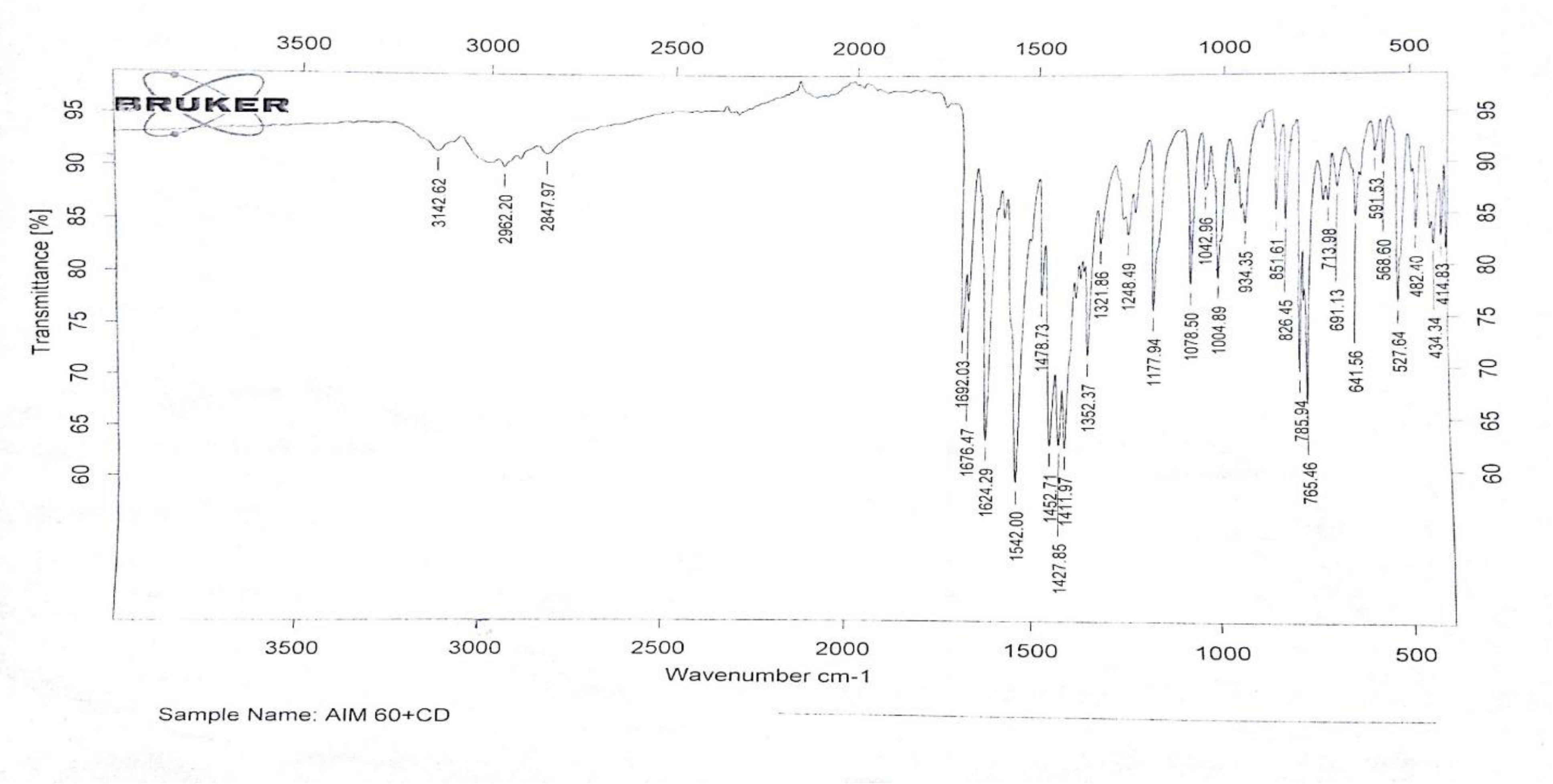

Figure S25. FTIR spectrum of Cd(II) Complex.

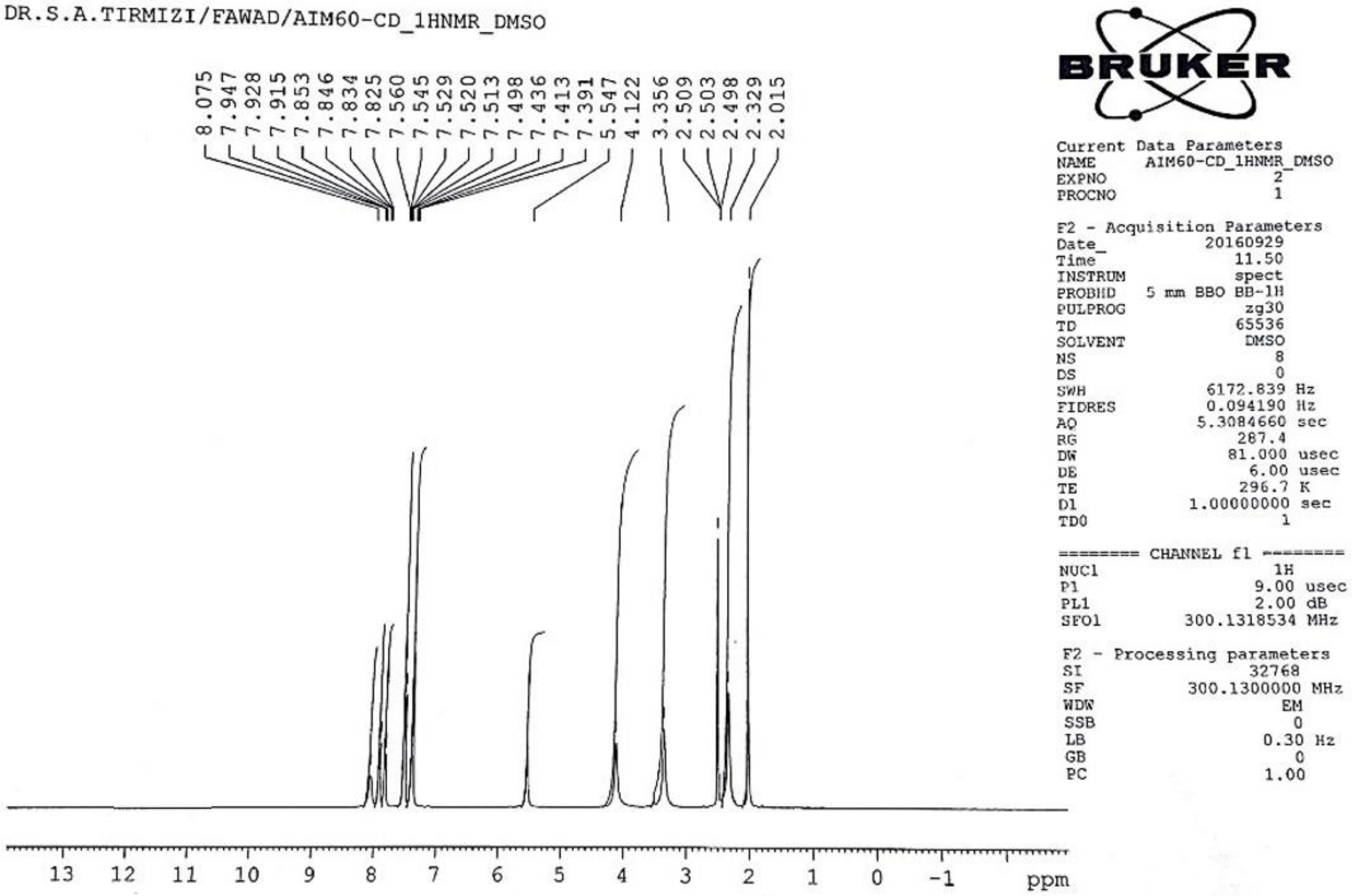

Figure S26. ^{1}H-NMR spectrum of Cd(II) Complex.

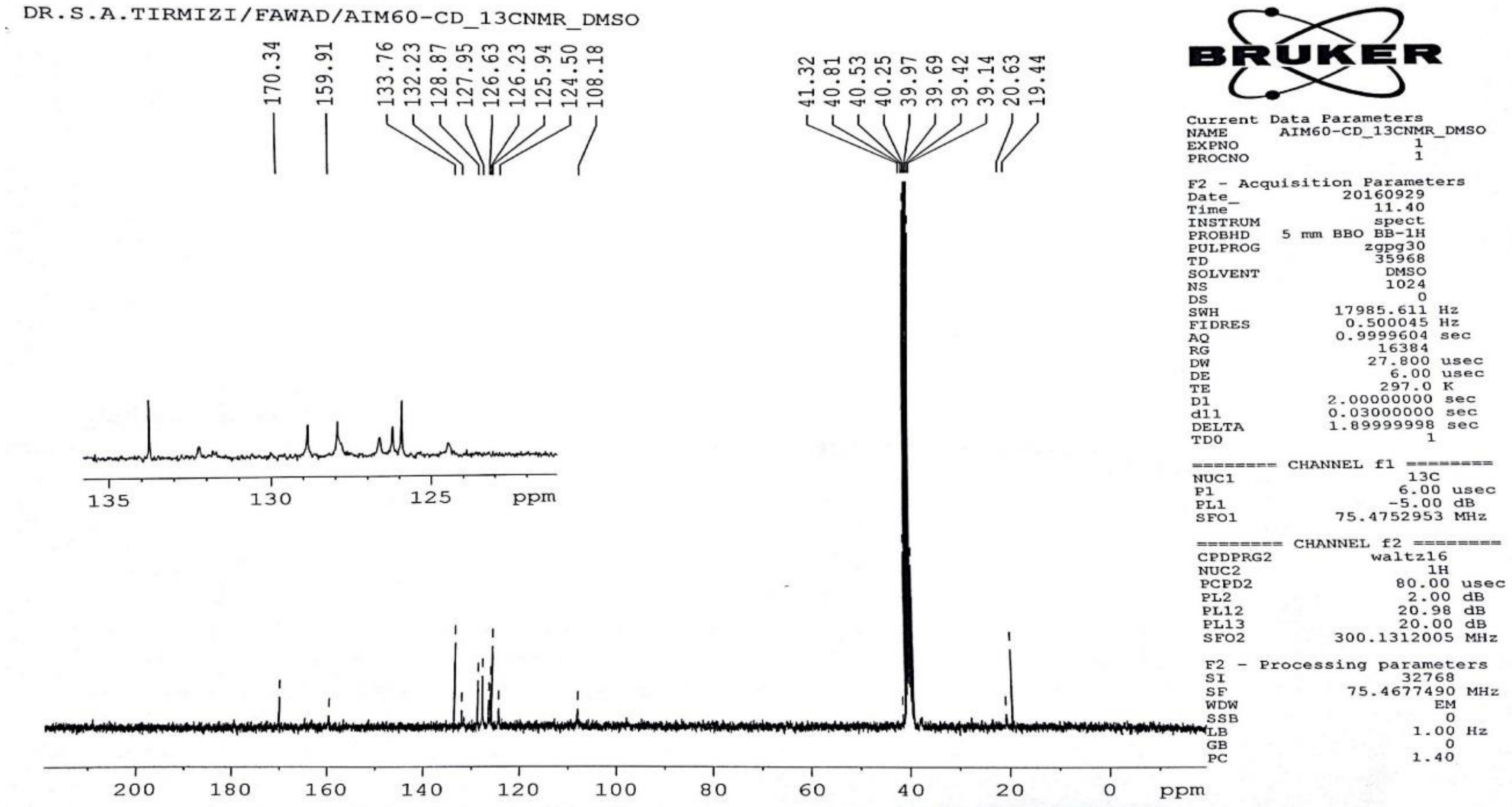

Figure S27. ^{13}C-NMR spectrum of Cd(II) Complex.

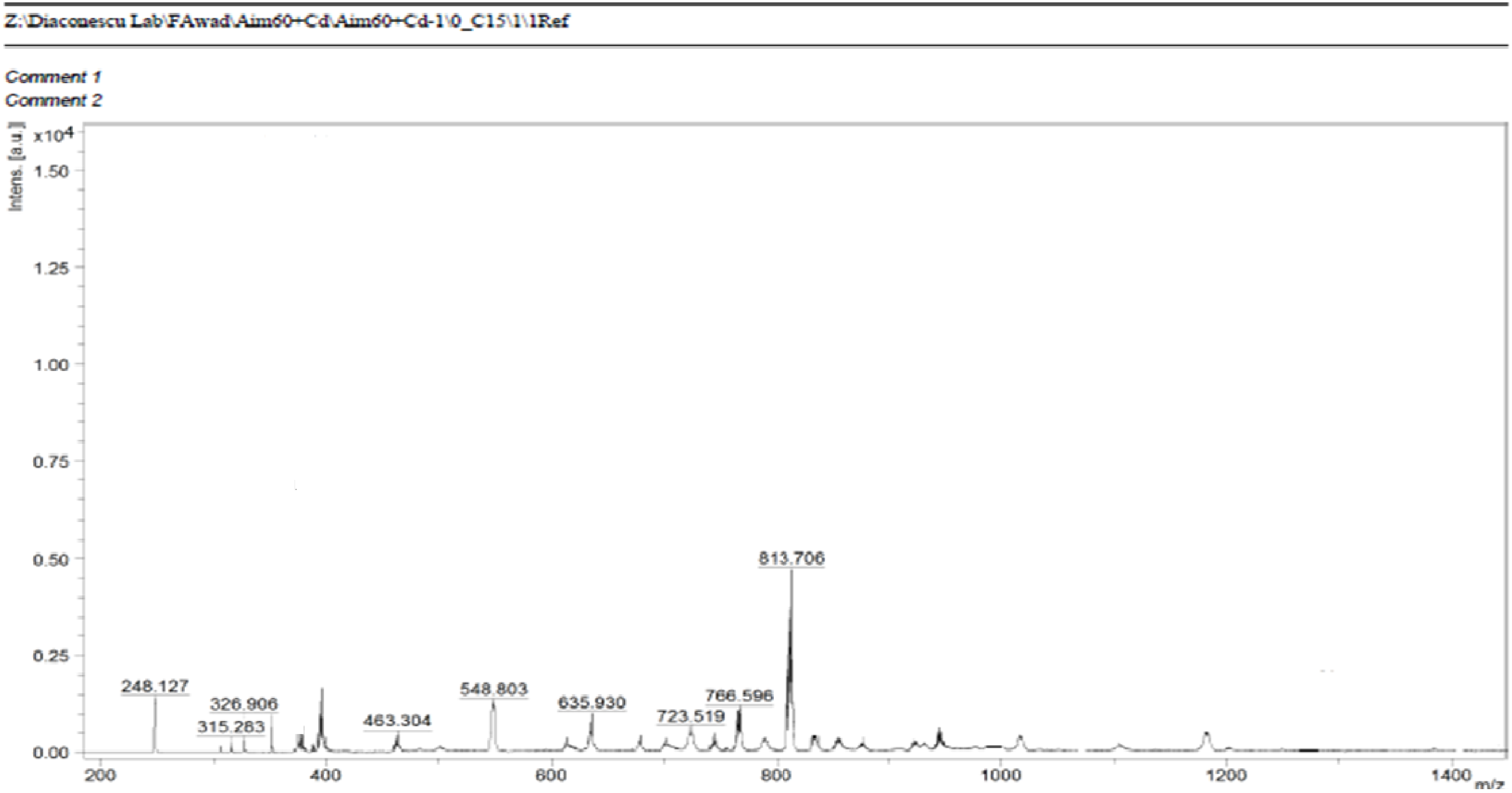

Figure S28. MALDI spectrum of Cd(II) Complex.

Table S1. Selected electronic spectral analysis of compounds.

Compounds	Conductance Ohm^{-1} cm^{2} mol^{-1}	Possible Transition	λ_{max} (nm)
Ligand	-	π–π*, n–π*, CT	325, 294, 284
Cu(L)$_2$	1.1	LMCT	337
Ni(L)$_2$	1.3	LMCT	337, 283
Co(L)$_2$	1.7	LMCT	322, 282
Zn(L)$_2$	-	CT	341
Pd(L)$_2$	1.1	LMCT	330
Cd(L)$_2$	-	CT	359
